# Mechanistic studies and therapeutic potential of angiopoietin in head and neck tumor angiogenesis

**DOI:** 10.3389/fonc.2025.1529225

**Published:** 2025-04-07

**Authors:** Xiaojuan Zhong, Yujie Fei, Haihui Zhao, Jiao Chen, Mingyu Gao, Yi Huang, Wei Fei

**Affiliations:** ^1^ School of Medicine, University of Electronic Science and Technology of China, Chengdu, Sichuan, China; ^2^ State Key Laboratory of Oral Diseases & National Center for Stomatology & National Clinical Research Center for Oral Diseases, West China Hospital of Stomatology, Sichuan University, Chengdu, Sichuan, China; ^3^ School of Stomatology, Southwest Medical University, Luzhou, Sichuan, China; ^4^ Yibin Second People’s Hospital, Yibin, Sichuan, China; ^5^ Department of Maxillofacial Surgery, Sichuan Provincial People's Hospital, School of Medicine, University of Electronic Science and Technology of China, Chengdu, China; ^6^ Department of Oral and Maxillofacial Surgery, Wenjiang Hospital, Sichuan Provincial People’s Hospital, Chengdu, Sichuan, China

**Keywords:** head and neck tumors, angiopoietins (Ang-1 and Ang-2), Tie receptors, tumor invasion, anti-angiogenic therapy (AAT)

## Abstract

Head and neck tumors represent a prevalent category of oral and maxillofacial malignancies, posing significant therapeutic and prognostic challenges due to their complex anatomical structure, tumor heterogeneity, and resistance to conventional therapies. Recent studies have highlighted the strong association between tumor progression and neoangiogenesis, with the angiopoietin (ANG) family playing a central role in this process. Comprising ANG1, ANG2, ANG3, and ANG4, these factors regulate multiple signaling pathways that promote cellular growth, differentiation, and proliferation, thereby driving angiogenesis and accelerating tumor growth and metastasis. Therefore, a comprehensive investigation of the ANG family’s role in head and neck tumors may offer critical insights into tumorigenesis mechanisms and unveil novel therapeutic targets. Such research has the potential to improve treatment outcomes and enhance the quality of life for patients.

## 1 Introduction

Head and neck cancer (HNC), as a member of head and neck tumors, is the sixth most common cancer in the world, causing over 450,000 deaths and 600,000 new cases each year (1, 2). Despite significant advancements in diagnostic and therapeutic approaches, the 5-year survival rate for most HNC subtypes remains below 50%. Poor prognosis is usually attributed to tumor heterogeneity, drug resistance and immunosuppression (3). And certain disease features are easily overlooked by patients at an early stage, ultimately leading to few effective methods for early detection and therapeutic drug development (4). The development of new drugs and therapeutic strategies remains a significant challenge. With the recent advances in medicine, it has become clear that tumor formation and progression are associated with the abnormal development of new blood vessels called neoangiogenesis.

**Figure 1:**
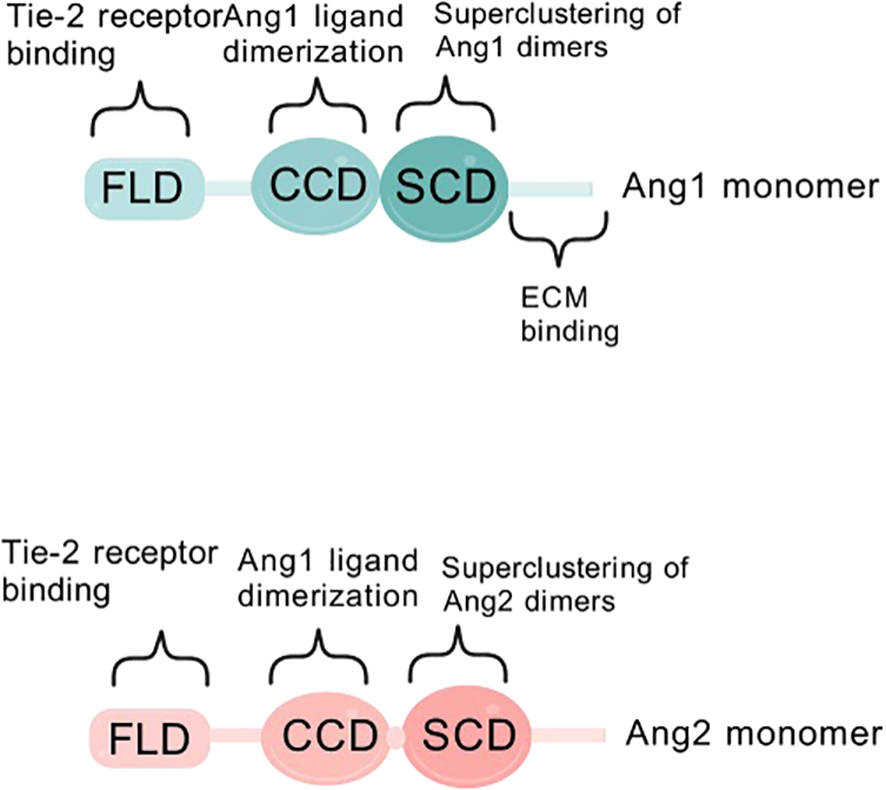
Schematic diagram of ANG1/ANG2 structural domains. Structural domains of angiopoietin-1 (ANG1) and angiopoietin-2 (ANG2). FLD is a fibrinogen-like structural domain, CCD is a coiled-coil structural domain, and SCD is a supercluster domain.

Angiogenesis is a broad term that includes endothelial cell migration, proliferation, tube formation, and intestinal ligamentation, as well as peri-EC recruitment and extracellular matrix formation (5), a complex multistep process regulated by interactions between angiogenesis inhibitors and growth factors (e.g. angiopoietin ANG, insulin-like growth factor IGF, fibroblast growth factor FGF, vascular endothelial growth factor VEGF) in the extracellular matrix (6). Tumor angiogenesis is regulated by angiogenic factors, some of the most patient angiogenic factors, such as vascular endothelial growth factor and ANG *in vivo*, are produced by macrophages and other immune cells in the tumor microenvironment (7, 8). The ANG family comprises small glycoproteins that play pivotal roles in blood vessel formation and repair. Members of this family include ANG1, ANG2, ANG3, and ANG4, which share a short amino-terminal structural domain for protein aggregation, a centrally located coiled-coil pattern, and a c-terminal fibrinogen structural domain. Notably, the fibrinogen-like domain is closely associated with the core coiled-coil motif via a short linker segment (9–11) (**Figure 1**).

In the tumor microenvironment, ANG2 synergizes with angiogenic factors, including VEGF, to promote late-stage angiogenesis (2). During tumor development, ANG family proteins play a key role in promoting invasion and progression of hypoxic tissues (e.g. solid tumors), while endothelial cell proliferation is driven by growth molecules. Although VEGF dominates angiogenesis, the Ang-Tie-2 pathway also has anti-tumor angiogenic potential (12, 13).

Despite significant advancements in tumor angiogenesis research, the role of ANG family in HNC remains an underexplored yet promising therapeutic target. While the VEGF pathway has long been the primary focus of angiogenesis studies, accumulating evidence indicates that the ANG-Tie axis plays a critical role in vascular stability, tumor microenvironment remodeling, and drug resistance, sometimes functioning independently of VEGF signaling. This review systematically integrates recent findings on ANG1, ANG2, ANG3, and ANG4, providing a comprehensive analysis of their distinct yet interconnected roles in tumor angiogenesis, invasion, and therapeutic resistance. Given that most studies have focused on VEGF-mediated regulation, the mechanisms underlying ANG signaling remain incompletely understood. By synthesizing recent advancements, we examine the impact of ANG on HNC progression, immune modulation, and resistance mechanisms, while exploring its potential as a target for anti-angiogenic therapy. This review aims to provide a comprehensive perspective to guide future research and facilitate the clinical translation of ANG-targeted therapies.

## 2 Association of ANG families with head and neck tumors

### 2.1 ANG family members and their biological properties

#### 2.1.1 ANG1

**Figure 2:**
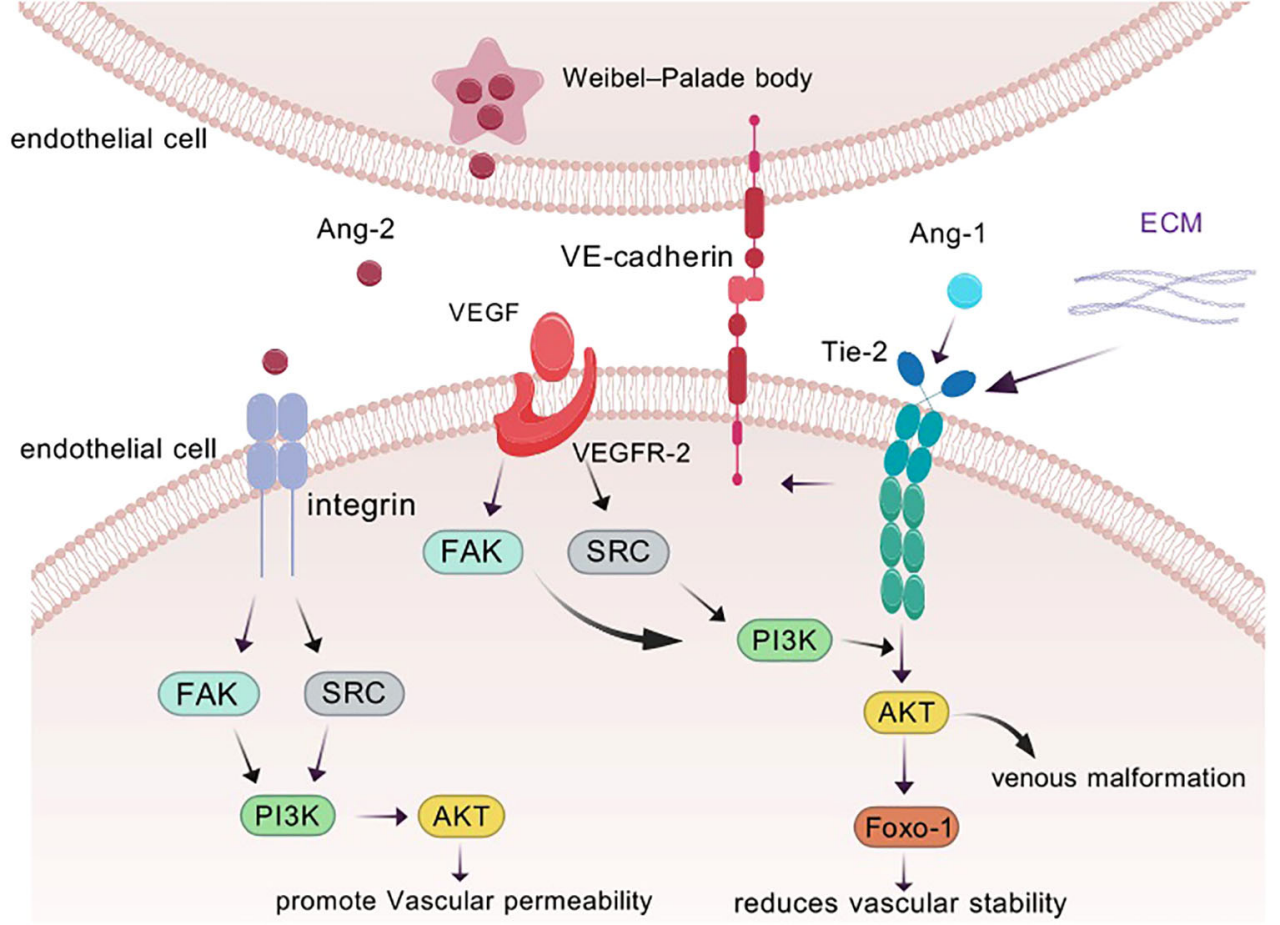
ANG1 is able to trigger Tie assembly at endothelial cell-endothelial cell (EC-EC) junctions as well as at endothelial cell-extracellular matrix (ECM) contacts. Binding of vascular endothelial growth factor (VEGF) to its receptor also modulates vascular permeability, reduces vascular stability, and promotes angiogenesis. In response to inflammatory factors, ANG2 is released from the Weibel-Palade bodies of endothelial cells and transmits different signals through binding to integrins. to promote increased vascular permeability.

Since 2003, researchers have gained insight into the characteristics of ANG1 as a member of the angiopoietin family, and its activation mechanism and subsequent biological effects have become a hot research topic. Specifically, the core of ANG1 activation lies in its precise targeting and activation of the Tie-2 signaling pathway, which is like a precise molecular key to unlock life. Firstly, ANG1 shows its power in multiple dimensions of inflammation with its excellent inhibitory ability, especially in the regulation of vascular permeability and inhibition of leukocyte adhesion on activated endothelial cells, which opens up new avenues for the treatment of sepsis, acute lung injury and other vascular permeability-related diseases (7). Under physiological conditions, in order to guard vascular homeostasis, ANG1 is maintained at a high concentration in the circulation, ensuring the stability and integrity of the vascular structure (14). In contrast, the serum of cancer patients shows a different picture, with a rise in ANG2 levels, a disruption of the ANG1 to ANG2 balance, and a significant decrease in the ratio, signaling vascular abnormaliTies in the disease state (15, 16). The activation of Tie-2 by ANG1 promotes the generation and survival of vascular endothelial cells. The cornerstone of this effect lies in its strengthening of the endothelial cell-extracellular connectivity (EC-EC) and the actin cytoskeleton, and especially in its stabilizing effect on vascular endothelial cadherin (VE-cadherin), which is an impenetrable defense for vascular stabilization, as it effectively protects against VEGF- or inflammatory cytokine-induced protein loss (17). Activation of Tie-2 also triggers an exquisite displacement of the receptor from the intercellular junction to the tail end of the cell, where it embraces the extracellular matrix (ECM), further deepening the connection between endothelial cells and the external environment (18). Delving deeper, ANG1 activates and cares for vascular endothelial cells through its dual strategy of anti-apoptosis and pro-proliferation, paving the way for vascular growth and repair. In this process, the Tie-2 receptor becomes the stage for ANG1 to work its magic, and the two work hand in hand to regulate the integrity and stability of blood vessels (**Figure 2**). ANG1 is essential for cardiovascular development during embryogenesis and for the maintenance and repair of the vascular network in adults. Its critical role in vascular biology is well-established (19). Notably, although ANG1 and ANG2 are structurally similar, differing only in a subtle binding displacement (**Figure 1**), this does not diminish their binding to Tie-2 or alter the rate of dissociation, and does not fully account for the differences in signaling between them (20). However, it is this subtle difference that confers ANG1 with unique physiological functions, such as promoting the migration of vascular endothelial cells and actively participating in the weaving and remodeling of neovascular networks. In the field of oncology, the overexpression of ANG1 has even demonstrated its potential to inhibit the invasion and metastasis of tumor cells, providing a new perspective to reduce the risk of distal tumor metastasis (21). In conclusion, ANG1, with its unique activation mode, plays a crucial role in angiogenesis, stability maintenance, inflammatory response regulation, and tumor metastasis inhibition in various physiological and pathological aspects, and its study not only deepens our understanding of vascular biology, but also opens up new avenues for the prevention and treatment of related diseases (22) (**Figure 2**).

#### 2.1.2 ANG2

ANG2, as an environmentally adaptable agonist, is unique in that it mainly resides in the Weibel-Palade vesicles of endothelial cells, waiting for the right moment. Its activation may be induced by inflammatory factors: when the cells are provoked by inflammatory factors, ANG2 is awakened and breaks out from these secretive vesicles to bravely take up the important task of counteracting the ANG1—TIE-2 signaling axis (23) (**Figure 2**). In stark contrast to the facilitating role of ANG1, ANG2 functions as an antagonist within the angiopoietin family, primarily regulated by endothelial cells during vascular remodeling. (ii) Signaling through integrin heterodimers (24), which in turn activates a series of downstream pathways that not only promote endotheli ANG2 disrupts the connection between endothelial cells and perivascular cells, promoting cell death and vascular degenerational cell proliferation and migration, but also quietly accelerate angiogenesis and tumor expansion (25, 26).

**Figure 3:**
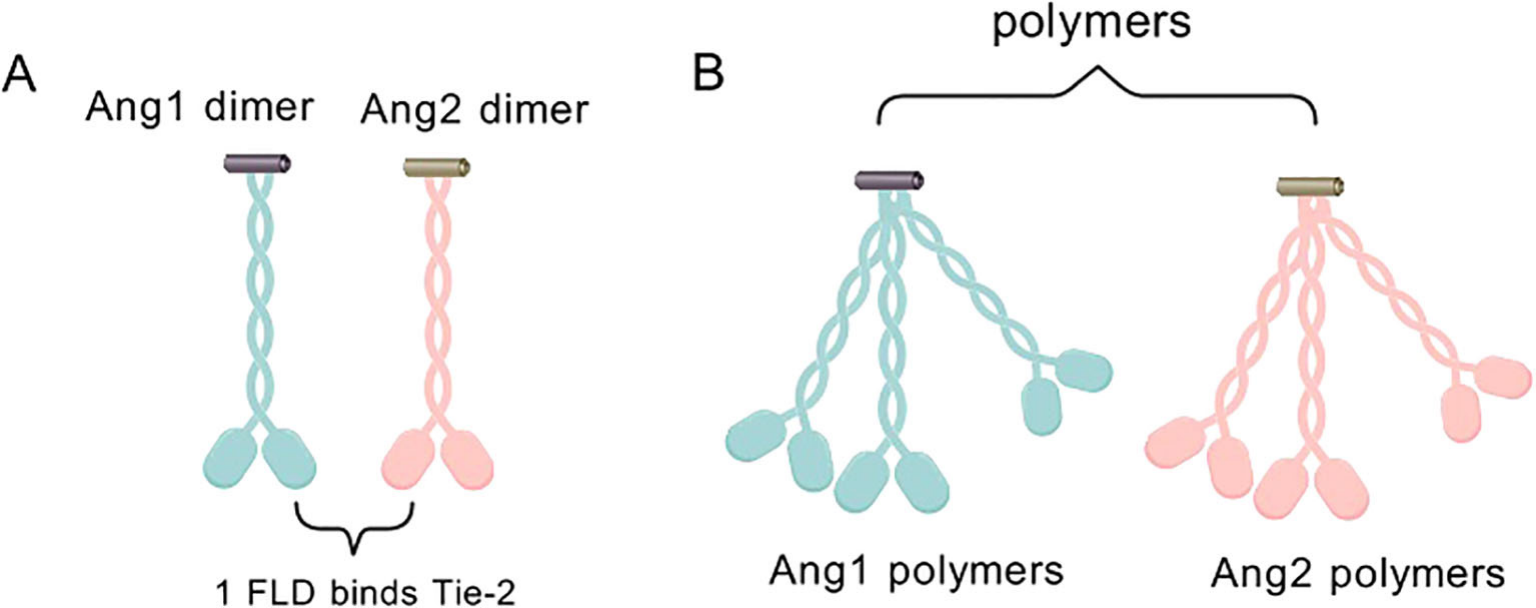
**(A)** ANG1 and ANG2 monomers combine to form an asymmetric dimeric structure, which is characterized by the presence of only one FLD capable of binding to Tie-2. **(B)** ANG1/ANG2 dimers can form multimers by dimerization on SCDs.

Deeper dissection of the molecular structures of ANG1 and ANG2 revealed that the two are like twins, sharing the c-terminal fibrinogen-like structural domain (FLD) that is in intimate contact with the Tie-2 receptor, but remaining detached from Tie-1 (**Figure 3A**).

**Figure 4:**
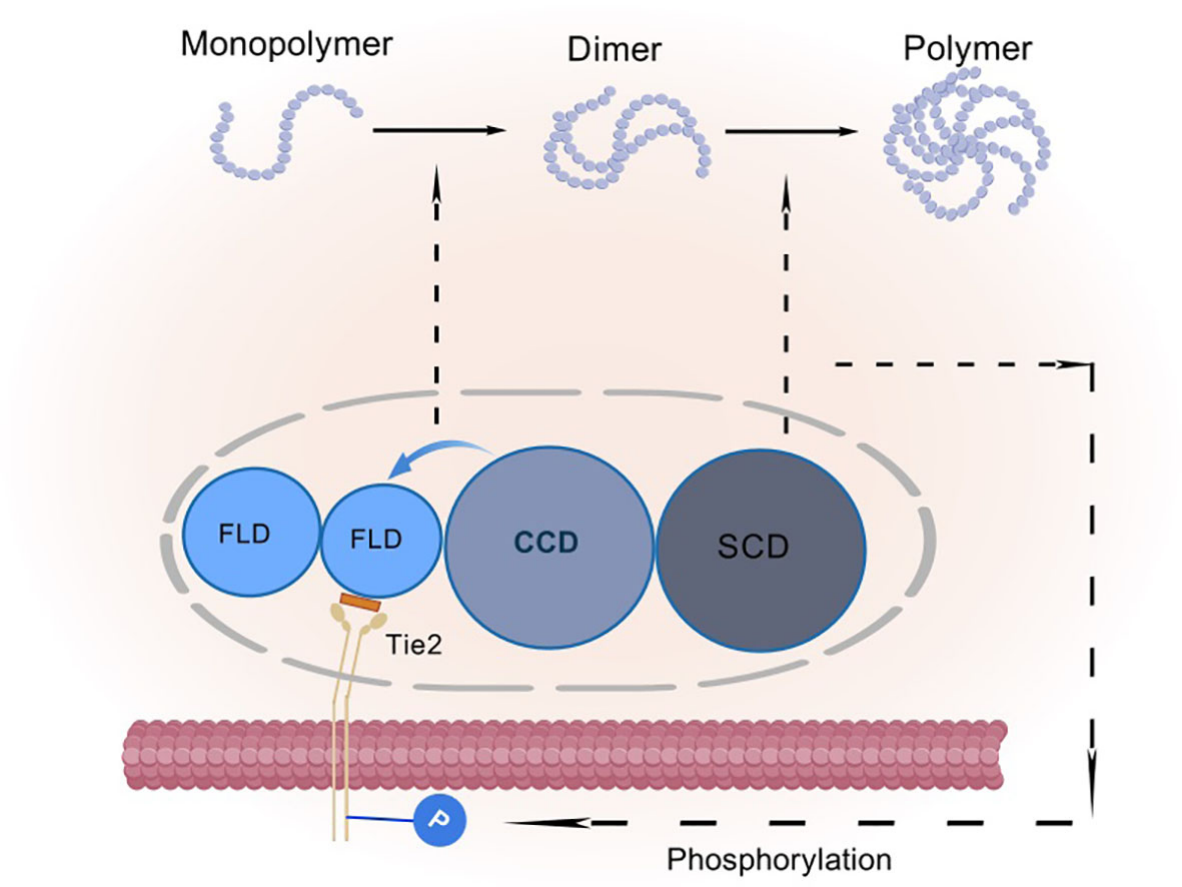
Schematic representation of angiopoietin activation of Tie-2 phosphorylation: FLD is a fibrinogen-like structural domain, CCD is a coiled-coil structural domain, and SCD is a superclass domain. CCD causes the Ang monomer to form an asymmetric dimer, resulting in only one FLD binding to Tie-2. SCD causes the dimer to aggregate into a heterogeneous multimer, which further facilitates the activation of Tie-2 phosphorylation.

They also cleverly weave the coiled-coiled structural domain (CCD) in the middle, which is not only a key enabler for them to form asymmetric dimers, but also ensures that only one FLD can be tightly embraced with Tie-2. In contrast, the supercluster domain (SCD), this brief n-terminal region, is their secret weapon for polymerization into complex multimers (**Figure 3B**) (27). In this delicate layout, the binding of FLD to Tie-2, the shaping of dimer asymmetry by CCD, and the catalysis of multimer formation by SCD combine to promote Tie-2 phosphorylation activation (28) (**Figure 4**).

ANG2 co-localizes with pro-angiogenic factors including VEGF in perivascular niches of malignant tumors. Endothelial-derived ANG2 induces vascular destabilization through Tie2 receptor modulation, while VEGF orchestrates three key pathophysiological processes: enhanced vascular permeability, neoangiogenesis, and lymphangiogenesis (6, 29, 30). Tumor-derived VEGF has been found to be a potent inducer of ANG2 expression in endothelial cells, thereby destabilizing the host vascular system. VEGF regulates vascular permeability, angiogenesis and lymphangiogenesis (31). And breast cancer VEGFR, as the faithful messenger of VEGF, is driving tumors such as breast cancer and malignant melanoma to the point of no return (26, 32–34). The potential impact of ANG2 on vascular destruction is well demonstrated in experiments with transgenic mice, in which impaired angiogenesis significantly leads to embryonic death when ANG2 expression levels are abnormally elevated. In particular, ANG2 plays a central role in tumor-induced angiogenesis during pathological angiogenesis. It contributes to tumor enlargement and increased risk of metastasis. Therefore, ANG2 has also been proposed as a biomarker for different cancer types. The expression levels of ANG2 are all proportional to cancer stage. However, further studies are needed to determine its exact role and expression levels in various types and subtypes of lung cancer. In a population-based study of patients with hepatocellular carcinoma, ANG2 was negatively associated with overall survival. ANG2 levels were upregulated in the liver compared to normal tissues, and these patients typically had higher ANG2 levels than the healthy population (35). Remarkably, in certain contexts, ANG2 can also swing into a direct agonist of the Tie-2 receptor, activating the Tie-2 signaling pathway. However, this ability is not an unconditional gift; in an arena where VEGF is absent, ANG2 turns to inhibit Tie-2 signaling, leading the vasculature towards degeneration (36).

#### 2.1.3 ANG3

ANG3, a member of the angiopoietin family, uniquely inhibits ANG1-induced Tie-2 phosphorylation. It also acts as a strong activator of the Tie-2 receptor, which is critical for vascular endothelial cell survival and migration. As a bioactive protein, ANG3 enhances the survival of human umbilical vein endothelial cells (HUVECs) but has minimal impact on their migratory behavior. The activation mechanism may proceed as follows: in mouse lung tissue, ANG3 is fully activated, significantly enhancing the phosphorylation of Tie-2 and Akt, demonstrating a stronger activation effect. In this context, ANG3 exhibits greater efficacy than ANG4 in promoting both survival and migration of mouse lung endothelial cells (37). Investigating the anticancer mechanism of ANG3, we observed that it functions as an inhibitor by suppressing the excessive proliferation and survival of endothelial cells, effectively blocking tumor neovascularization, and thereby impeding tumor growth and metastasis. This mechanism is mediated by ANG3’s specific targeting of ANG1 and the VEGF-driven Erk1/2 and Akt kinase activation pathways. Through this signaling intervention, ANG3 significantly reduces tumor blood supply, leading to nutrient deprivation and growth inhibition in tumors (38).

#### 2.1.4 ANG4

ANG4, another member of the angiopoietin family, is known for its high-profile expression in the human lung. It inherits the qualities of ANG1 and becomes a great fan of the Tie-2 receptor, activating its potential ability to (39). ANG4 is even more unique in the field of angiogenesis, where it plays an indispensable role as a counterbalance to the angiogenic wave set off by recombinant growth factors with its remarkable inhibitory talents. Unlike some simplistic anti-cancer methods, ANG4’s inhibitory action does not rely on a direct toxic attack on tumor cells, but rather takes a smarter path - hitting the lifeblood of angiogenesis head-on. In the precision-designed experiments, despite the ubiquitous presence of ANG4, the proliferation rate of the tumor cells did not seem to be affected in the slightest, and they continued to multiply normally. This result clearly reveals that ANG4 is characterized by the network of blood vessels that power tumor growth, rather than the tumor cells themselves.

This discovery has undoubtedly opened up a whole new line of therapeutic thinking. ANG4 inhibits tumor angiogenesis via dual mechanisms: Tie2 receptor blockade and pericyte-mediated vascular pruning, reducing microvessel density and interstitial pressure in preclinical models. Its selective suppression of pathological neovascularization preserves physiological angiogenesis in chronic inflammation. Current research focuses on ANG4-VEGF crosstalk and spatiotemporal therapeutic optimization, advancing precision anti-angiogenic strategies (40). The study of ANG4 has been conducted in a number of countries.

### 2.2 Ang receptors

#### 2.2.1 Tie-1

Tie-1 (tyrosine kinase with immunoglobulin-like and EGF-like domains-1), as a member of the tyrosine kinase family with immunoglobulin-like and EGF-like domains, occupies an indispensable position in the biological activities of vascular endothelial cells. It is activated in the following 2 ways: (a) in close collaboration with integrins: the mechanism of activation is subtle in its close collaboration with integrins to promote angiogenesis. (b) Tight binding with ANG1 ligand: When Tie-1 binds tightly with ANG1 ligand, a story about the fate of blood vessels will quietly appear. This binding is not just a simple touch between molecules, but also activates a series of downstream signaling pathways, which work in concert to finely regulate the formation and stability of blood vessels, ensuring that each pathway is unobstructed (41, 42).

The role of Tie-1 is much more than a bystander or helper; it is the master regulator of vascular endothelial cell function and a key driver in the process of vascular development. Under its command, endothelial cells seem to gain unlimited vitality, promoting proliferation and migration as well as angiogenesis. At the same time, Tie-1 also strengthens the defense of the blood vessel wall with its unique charisma, making it more impregnable. Tie-1’s ability is far more than that. It is also a regulator, able to acutely sense and respond to subtle changes in vascular permeability, adjusting its strategy in time to ensure that the fluid balance is not disrupted. In the storm of inflammation, Tie-1 stands up to the storm and builds a solid defense for the vascular system with its robust regulatory ability, protecting the body from unnecessary damage (43, 44).

#### 2.2.2 Tie-2

**Figure 5:**
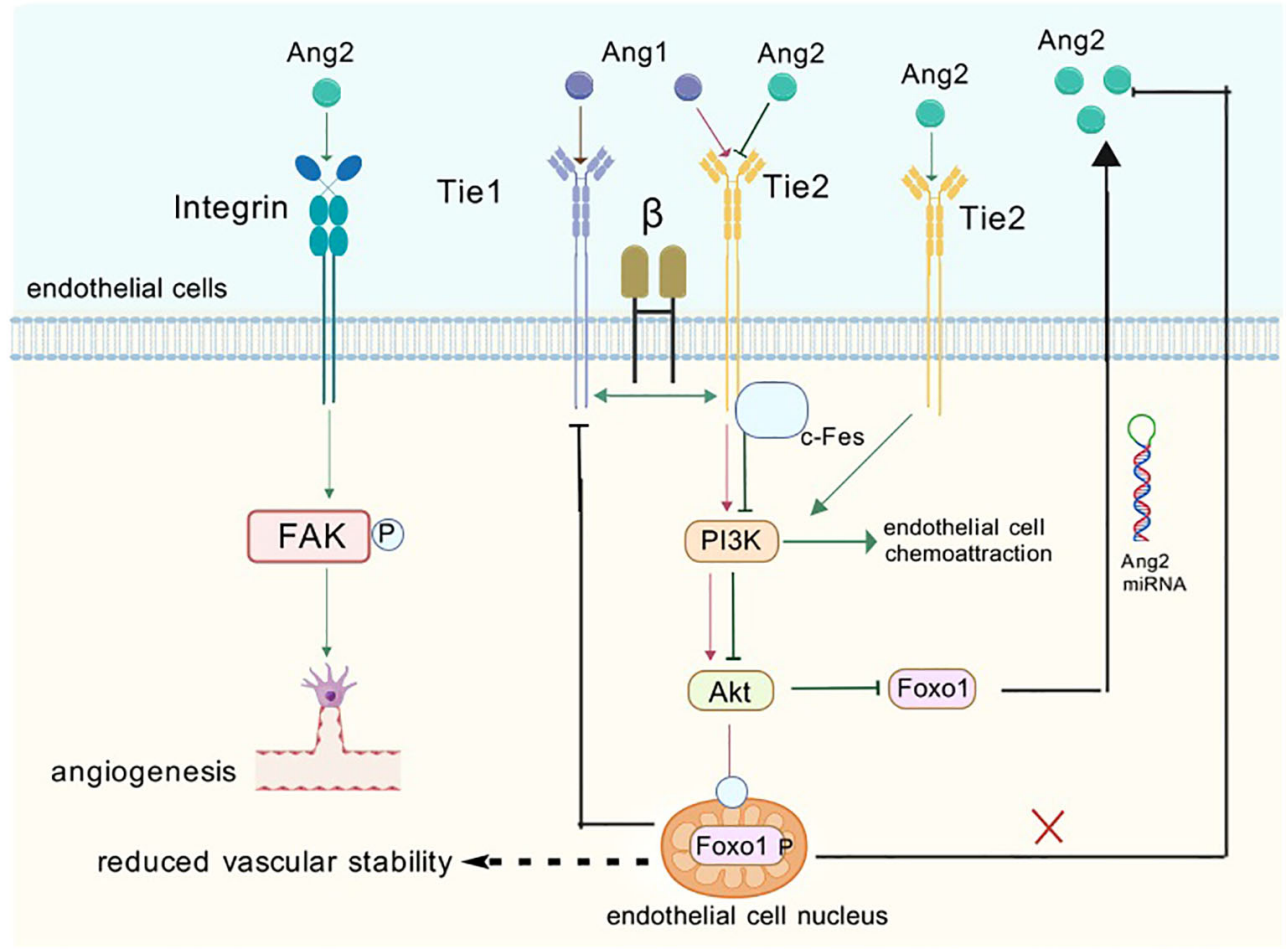
When ANG1 and 2 bind to endothelial cells they activate a number of signaling pathways. ANG1 activates downstream signaling pathways when it binds to the receptor, which promotes endothelial cell value-addition and activation while also inhibiting the formation of ANG2. ANG2 has a dual role, acting as an antagonist as well as an agonist of Tie-2. This means that ANG2 can interact with Tie-2 receptors to produce different biological effects. When ANG2 binds to integrin-forming complexes, it can activate down-game signaling pathways that induce endothelial cell migration and bud formation, which are further involved in the process of angiogenesis and remodeling.

Tie-2, as another important member of the tyrosine kinase receptor family, has its life’s music closely revolving around the growth and differentiation of vascular endothelial cells. It acts as a bridge between the world of angiogenic factors and the fate of endothelial cells, and ensures the harmony and stability of the vascular system through a delicate regulatory mechanism. Its activation mechanism is similar to that of Tie-1: (a) Tight binding to ANG1 ligand: ANG1, the companion of Tie-2, with its strong agonistic effect, provides a solid backbone for the survival of endothelial cells, the stability of blood vessels, and the maintenance of endothelial barrier function (45). When ANG1 binds to Tie-2, a complex intracellular signaling process is initiated; the interaction between ANG1 and Tie-2 energizes the serine kinase AKT, which in turn leads to a series of downstream signals. This river of signals ultimately inhibits the growth of Forkhead box protein - Tie1 (FOXO1) and indirectly stifles FOXO1’s tendency to repress the ANG2 gene (46, 47) (**Figure 5**). (b) Through activation of the AKT pathway: activation of the AKT pathway, like a fuse, triggers phosphorylation of Foxo1. In this process, like a precise mechanical bonding, Foxo1 is led to the edge of the cytoplasm and its function is inhibited, while ANG2 expression is silently reduced in this delicate balance. However, when PI3K is inhibited, the path of AKT activation is blocked and the phosphorylation of Foxo1 comes to an abrupt end. Lost in bondage, Foxo1, as if breaking free of gravity, returned to the interior of the cell nucleus. Here, it found its strength and activated its potential, which in turn drove a significant increase in ANG2 mRNA levels (48, 49) (**Figure 5**). (c) Through interactions between the structural domains of Fn3: it was found to signal its binding to ANG1 (50) and its activation was also achieved through interactions between Fn3 structural domains. These interactions form a dimeric structure that promotes the activation and phosphorylation of Tie-2 (51) More interestingly, Tie-1 and Tie-2 are not independent of each other. They interact with each other and jointly regulate the effect of the Ang family on Tie-2 phosphorylation (52) (**Figure 5**). This finding not only e1-nriches our understanding of the relationship between the Tie receptor families, but also provides new clues for us to explore the deeper mechanisms of angiogenesis and stabilization.

### 2.3 Relationship between ANG families and tumor angiogenesis

#### 2.3.1 Angiogenesis and tumor progression

When the tumor continues to develop with active angiogenesis, the tumor cells may secrete pro-angiogenic factors, and when the pro-angiogenic factor signals are dominant, blood vessel formation will be induced, a process known as the “angiogenic switch”. This process is called the “angiogenic switch”, which is the initiating mark of tumor vascularization. The angiogenic switch can make the tumor active from the dormant state, and when the tumor vascularization is initiated, an abnormal vascular network will be formed, leading to impaired tumor perfusion, creating a hypoxic microenvironment, which can promote the invasiveness of tumor cells, and at the same time, it can hinder the killing effect of the immune cells on the tumor cells, which can further promote the tumorigenesis (53, 54).

The formation of new capillaries, termed “angiogenesis”, is one of the most widespread and fundamental biological processes in mammals. Angiogenesis is an important event in a variety of physiological environments, such as embryonic development, chronic inflammation, and wound repair, and angiogenesis is a feature of a limited number of physiological processes; the etiology and pathogenesis of a growing number of pathological conditions have been shown to be the result of angiogenic responses. Ocular diseases, vascular malformations, and tumors are a few examples where angiogenesis plays an important role (55–57).

#### 2.3.2 ANG family regulation and tumor angiogenesis

The ANG family (ANG1, ANG2, and ANG4) primarily mediates its biological functions via Tie-2 receptor interactions on endothelial cells (58). The absence or overexpression of ANG can lead to impaired interaction between endothelial cells and perivascular cells, which in turn affects the structural integrity of the vasculature (59–61). The ANG have been shown to have a significant impact on the structural integrity of the vascular system. Studies have shown that ANG1, ANG2, and ANG4 activate PI3-AKT signaling pathways, which promote cell growth, differentiation, and proliferation, thereby promoting angiogenesis (62, 63). ANG2 and ANG4 activate PI3-AKT signaling pathways, which promote cell growth, differentiation and proliferation, thereby promoting angiogenesis (57, 58). In recent years, ANG2 has been intensively investigated as a second-generation anti-angiogenic candidate molecule, as it plays a key role in both agonism and antagonism as an important ligand for the maintenance of the resting state of the endothelial cell ANGPT1/Tie-2 signaling axis. In preclinical studies, deletion of the Angpt2 gene results in a transient delay in primary tumor growth (64). In postoperative adjuvant therapy, the combination of ANGPT2 neutralizing antibody and low-dose beat-to-beat chemotherapy not only inhibits the angiogenic response of endothelial cells within the metastatic site, but also suppresses their inflammatory response, thus limiting metastasis (65). These findings suggest that ANG have broad and far-reaching effects on the vascular and lymphatic systems, and that there are both complementary and potentially contradictory points between them and other important growth factors such as VEGF. Through these complex interactions, ANG play an integral role in several aspects of angiogenesis, permeability regulation, and lymphatic system development (66).

In addition to this, findings suggest that in addition to tumor growth and angiogenesis, systemic ANG2 overexpression promotes tumor lymphangiogenesis, lymph node and lung metastasis (67). Another study found that a higher proportion of tumor cells expressed ANG2 in patients with metastatic melanoma compared to patients with primary tumors and benign nevi (68). Further experimental analyses showed that ANG2 secreted by tumor cells does not affect the function of blood vessels or immune cells, but rather promotes the metastatic and colonization ability of tumor cells by controlling their metabolic functions. ANG2 secreted by tumor cells promotes melanoma metastasis and colonization ability by regulating the metabolic function and mitochondrial function of tumor cells (65). In various cancers, including hepatocellular carcinoma, researchers have identified a link between angiogenic stimulation and the epithelial-mesenchymal transition process (EMT). Studies by Dong et al, Ribatti et al. have demonstrated that EMT plays a key role in driving tumor progression by increasing invasion and metastasis (69). In some *in vitro* cancer models, such as melanoma, cervical and breast cancers, EMT markers (i.e., e.g., N-cadherin, Vimentin, Snail, and Twist) act as facilitators of cell motility and increase resistance to anticancer therapy (70). In addition, recent studies have highlighted the clinical significance of EMT in HCC and CCA. This includes its potential as a therapeutic target for cancer, as well as its role as a prognostic marker, the presence of which provides valuable insights into cancer therapy. In conclusion, ANG is important in tumor anti-angiogenic strategies in head and neck tumor therapy, and inhibition of the activity of molecules such as ANG2 shows potential therapeutic benefits (71).

**Figure 6:**
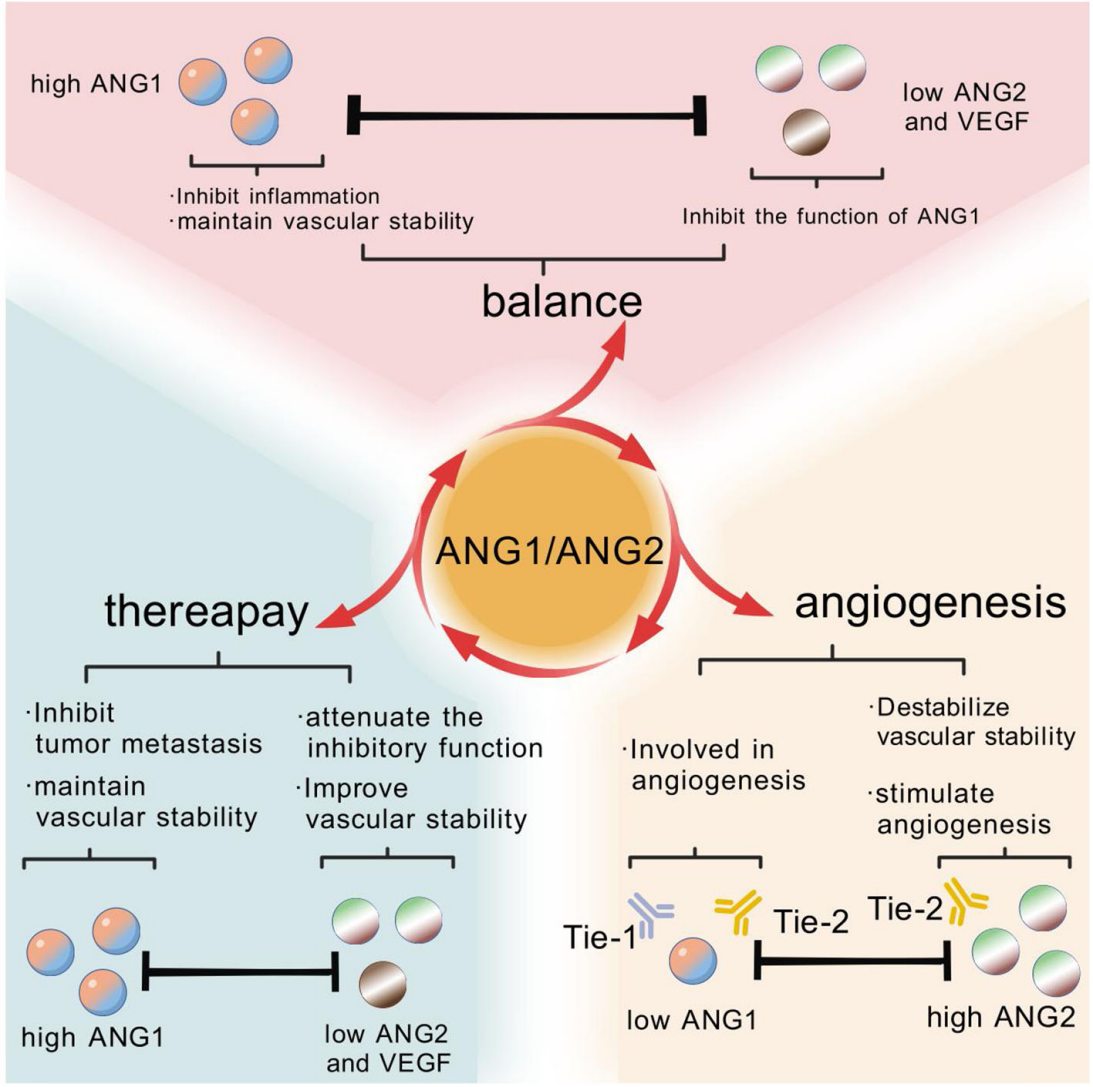
In the healthy state, the stable activity of vascular endothelial cells benefits from the balance maintained by the high expression of ANG1 and the low expression of ANG2 and VEGF. In a variety of cancer cases, this balance is disrupted by overexpression of ANG2 and a decrease in the ANG1 to ANG2 ratio, i.e., a rise in the ANG2/ANG1 ratio. Fortunately, after receiving anti-angiogenic therapy, the expression levels of ANG1, ANG2 and VEGF gradually normalize, suggesting that treatment helps to restore this important balance of cellular activity.

ANG2 disrupts endothelial-perivascular interactions, inducing cell death and vascular degeneration. However, together with VEGF, ANG2 promotes neointimal formation. Thus, angiopoietins play a crucial role in the angiogenic switch during tumor progression, and elevated expression of ANG2 relative to ANG1 in tumors correlates with poor prognosis. The central role of the angiopoietin/Tie signaling pathway in the regulation of physiological and pathological angiogenesis makes it an attractive target for the treatment of vascular disease and cancer (72). In addition, bone marrow (bm)-derived pro-angiogenic and/or tumor-infiltrating cells, including monocytes, macrophages, granulocytes and neutrophils, as well as specific cell subpopulations such as tumor-associated macrophages (TAMs), TIE-2-expressing monocytes (TEMs), and myeloid-derived suppressor cells (MDSCs) have been shown to provide high levels of angiogenic factors by locally promoting tumor angiogenesis in a paracrine manner (72). In the complex network of tumor angiogenesis, tam is recognized as a key driver of tumor angiogenesis (73). Whereas TEM, as a specific population of cells present in the blood, once recruited to the tumor microenvironment, they undergo a rapid process of differentiation and polarization, transforming into functional tam (74). Further, when these TEMs are eliminated within the tumor, their contribution to tam generation is blocked, thereby inhibiting the process of tumor angiogenesis (75). Compared to Tie-2-negative monocytes, circulating TEMs exhibit a higher angiogenic potential before they have even entered the tumor microenvironment. These cells have been pre-programmed in the circulatory system to express higher levels of pro-angiogenic genes, thereby promoting the angiogenic process within the tumor more efficiently (**Figure 6**).

In summary, ANG may play a two-fold role in tumor angiogenesis or inhibition, while the study of ANG function is dominated by ANG1 and ANG2. More unknown functions of ANG3 and ANG4 remain to be discovered by subsequent scholars.

## 3 Impact of ANG families on the treatment of head and neck tumors

### 3.1 Conventional treatments for head and neck tumors and their limitations

One of the subtypes of head and neck tumors are invasive tumors from soft tissues, glands and bones (including invasive tumors in the oral cavity range), each of which has distinct clinical, histological and molecular features (76). Their treatment is complex and delicate, and it involves a wide range of technological tools and individualized therapeutic strategies. In general, the choice of treatment depends on the type, size and location of the tumor as well as the overall health of the patient. For benign tumors, such as cysts or small, substantial tumors in the oral cavity, surgical excision is usually the treatment of choice. Through delicate surgical operations, the surgeon is able to remove the tumor completely while preserving the function and form of the surrounding normal tissues as much as possible. Post-surgery, patients typically undergo rehabilitation, including oral hygiene, wound care, and dietary adjustments. For malignant tumors, treatment is more complex and personalized, often requiring a multimodal approach beyond surgery, such as radiotherapy, chemotherapy, or traditional Chinese medicine. Radiotherapy uses high-energy radiation to target tumor cells, while chemotherapy employs drugs to inhibit tumor growth and metastasis. Treatment plans are tailored to individual patient profiles to maximize efficacy, minimize side effects, and improve both survival and quality of life (77).

However, current treatment modalities have limitations. While radiotherapy can rapidly inhibit lesion progression, its lower safety profile, significant side effects, and patient compliance issues restrict its efficacy. Additionally, radiotherapy alone often fails to fully eradicate lesions, leading to a high recurrence rate in patients (78). Surgical treatment aims to maximally remove tumors but often requires complex reconstructive procedures, such as pectoralis major myocutaneous flaps, forearm flaps, buccal fat pads, or anterolateral thigh flaps, to repair tissue defects. These interventions, while effective, can result in facial and cervical deformities, tissue loss, and psychological impacts, necessitating further postoperative rehabilitation (79). The elderly patient population warrants special attention due to age-related risks, including hypertension, diabetes, cardiovascular diseases, and other comorbidities. Surgical treatment and postoperative reconstruction in this group are associated with increased operative risks, prolonged procedures, and higher rates of complications, complicating clinical management (80). The length of the surgery and postoperative repair and reconstruction is long, and there are many postoperative complications, which makes the treatment more difficult. Postoperative cognitive dysfunction may be more likely to occur in elderly patients due to longer ICU stays (80). The patient may be more susceptible to postoperative cognitive impairment in the elderly (70). Additionally, age-related decline in respiratory function, including weakened cough reflexes, reduced mucus clearance, and degeneration of mucous glands and cilia, compromises airway defense mechanisms. These changes increase the risk of aspiration pneumonia and postoperative respiratory infections in elderly patients (81, 82). This has led to the development of respiratory infections in some elderly patients (71, 72). Psychological factors are equally critical. Oral cancer patients endure surgical and radiotherapy-related pain, compounded by high treatment costs, often leading to severe negative emotions such as depression. These psychological burdens adversely affect quality of life, morbidity, and mortality. Elderly patients, due to poorer physical and mental resilience, are particularly susceptible to such psychological disorders (83). The elderly patients have poorer psychological and physical qualities and are more prone to psychological disorders than younger patients (73).

In conclusion, the treatment of head and neck tumors is diverse and individualized, while all these treatment modalities have certain drawbacks. Elderly patients are more difficult to treat because they are often accompanied by other geriatric diseases, and they are prone to respiratory tract infections and psychological problems after surgery. Therefore, when treating head and neck tumors, it is necessary to take into account the patient’s specific situation, develop a personalized treatment plan, and pay attention to the patient’s psychological and social support. This set of problems also raises the relative difficulty of treatment.

### 3.2 The ANG family as a target for head and neck tumor therapy

The concept of anti-angiogenesis was first proposed in 1971 by Judah Folkman, who hypothesized that inhibiting neovascularization in the early stages of cancer development would stop tumor growth and metastasis and keep tumors dormant (84). Based on extensive literature, it has been established that inhibition of angiogenesis is an effective strategy to limit tumor growth in animal models carrying various cancers. To date, over 300 anti-angiogenic molecules have been identified as potential drug candidates, including many natural and synthetic compounds (56). Among targeted therapies against angiogenesis, adjuvant anti-ANG2 therapies may exhibit higher success rates compared to advanced metastatic therapies (85) Adjuvant anti-ANG2 therapies Considering the role of ANG2 in angiogenesis, it may be a more desirable target than VEGF. Especially in the absence of surgical intervention, micrometastases are not entirely dependent on VEGF-driven neoangiogenesis, and these make ANG2 a more critical therapeutic target (86–88).

#### 3.2.1 Key targets for ANG therapy

In recent years, progress has been made in anti-angiogenic therapy for prostate cancer and other types of solid tumors (89–92), ANG is not only involved in tumor angiogenesis, but in addition to its angiogenic activity, angiopoietin also plays a role in cancer cell proliferation, and thus may be an effective molecular target for cancer treatment (93). This has aroused great interest in the therapeutic role of angiogenic factors in head and neck tumors.

##### 3.2.1.1 ANG2/Tie-2 pathway targeted therapy

Researchers have found that treatments that inhibit neoangiogenesis by interfering with signal transduction pathways that regulate angiogenesis and growth or by directly targeting tumor-associated endothelial cells have been proposed as promising strategies (94) ANG2 expression has been tightly linked to tumor invasion and metastasis in a variety of human cancers, a link that is clearly far from being limited to its angiogenesis-promoting function, but involves more complex biological mechanisms (55) The ANG2/Tie-2 signaling system is essential for vascular development and function (95) The ANG2/Tie-2 signaling system is essential for vascular development and function (86). It has been shown that ANG2 can promote angiogenesis by activating Tie-2 receptors, or it can exert its proangiogenic effects in a Tie-2-independent manner. This means that even without the involvement of Tie-2, ANG2 can still promote angiogenesis by activating integrins. Therefore, inhibiting ANG2 activity may be beneficial for tumor therapy (96). In contrast, the expression of both ANG2 and VEGF is upregulated under hypoxic conditions. This suggests that in some types of cancer, other angiogenic factors besides VEGF are involved in the angiogenic process of tumors (97). In mouse models of glioma *in situ*, ANG2 inhibition significantly reduced tumor volume and decreased microvessel density, an effect that was unaffected by whether VEGF was also inhibited. When we simultaneously inhibited ANG2 and bound Tie-2 receptors, we observed an even more dramatic reduction in tumor volume (98, 99).

##### 3.2.1.2 Drug-targeted therapy

In HNSCC with high recurrence and low chemotherapy tolerance, the mechanisms of occurrence, progression, invasion, spread and metastasis have been progressively clarified and involve multiple steps similar to other solid tumors. HNSCC exhibits high vascular density with active lymphatic drainage, establishing tumor angiogenesis as a critical therapeutic target. Clinically available angiopoietin (ANG) inhibitors demonstrate dual anti-tumor mechanisms: (a) blocking ANG-mediated neovascularization through Tie2 receptor blockade, and (b) suppressing compensatory pro-angiogenic factors (VEGF, FGF2). These characteristics address HNSCC’s clinical challenges - multifocal primary lesions, hypervascularization, and frequent unresectability of primary tumors - positioning anti-ANG therapies as optimal candidates for localized treatment regimens (100). Researchers found that AMG 780, a monoclonal antibody, possesses the ability to inhibit the binding of ANG1 and ANG2 to the Tie receptor. Doses of AMG 780 ranging from 0.1 mg/kg to 30 mg/kg were given to patients by intravenous injection. The results showed that even at doses up to 30 mg/kg, no limitation of the maximum tolerated dose was observed. Therefore, for patients with solid tumors, the recommended dose to administer is 30 mg/kg. AMG780 has demonstrated significant efficacy when used alone or in combination with other chemotherapeutic agents. However, treatment with anti-ANG agents may result in a number of side effects, including anorexia nervosa, hypoalbuminemia, fatigue, and peripheral edema, and should be chosen with caution in clinical practice (101, 102). The clinical management of this drug should be done with caution (92, 93). Similarly, drugs such as bevacizumab have been successfully targeted against VEGF, and treatment with bevacizumab has shown significant results in not only inhibiting angiogenesis, but also effectively reducing the disease burden, as verified by a number of extensive preclinical and clinical data. However, the side effects and resistance of the treatment remain unclear (103–106).

Backed by solid preclinical data, researchers have successfully developed several therapeutic agents specifically targeting the ANG2-Tie-2 pathway. Among them, Trebananib (AMG386), an innovative ANG1/2-neutralizing peptidomimetic, is able to effectively block the interaction between the ligand and the Tie-2 receptor. This breakthrough drug has been rigorously evaluated in multiple early-stage clinical trials, and its safety and potential efficacy have been fully validated. The development of Trebananib not only provides a new strategy for the treatment of related diseases, but also demonstrates the great potential of drug design targeting the ANG2-Tie-2 pathway (107, 108). The development of neamine In contrast, a compound called neamine inhibits the action of angiopoietins, thereby inhibiting tumor angiogenesis and cancer cell proliferation. This suggests that targeting drugs to inhibit angiogenesis may be an effective strategy for oral cancer treatment (109). In the development of drugs targeting the ANG2-Tie-2 pathway, ribasitinib, a novel drug, not only significantly reduced Tie-2-mediated capillary formation in endothelial cell lines, but also effectively attenuated transient-dependent tumor cell endocytosis through endothelial cells. In a clinical trial in a mouse model of breast cancer, treatment with ribasitinib significantly reduced the growth rate of primary breast cancer and successfully reduced the development of lung metastases (110) Considering its inhibitory activity as an inhibitor of a variety of kinases, including Tie-2, we can speculate on its potential role in the future treatment of head and neck tumors.

Although preclinical studies have demonstrated that combining ANG2 and VEGF blockade significantly enhances antitumor effects compared to monotherapy, the off-target effects of VEGF inhibitors in existing combination regimens may compromise treatment specificity, a critical bottleneck particularly evident in vascular-targeted therapies for head and neck cancer (HNC). Circulating endothelial cells (CECs) and endothelial progenitor cells (EPCs) have been proposed as non-invasive surrogate biomarkers of angiogenesis in cancer and other diseases (111). CECs are defined as cells that enter the bloodstream and express endothelial markers in the absence of progenitor and hematopoietic markers. Within this theoretical framework, monitoring CECs offers unique clinical value. More importantly, flow cytometric detection of these quantitative indicators provides a molecular scale for optimizing the timing of combination therapies: it not only explains the biphasic fluctuations of CECs induced by chemotherapy combined with anti-angiogenic agents but also distinguishes the biological differences between pathological leakage and therapeutic response (112). This enables the construction of a “dynamic monitoring-mechanistic analysis-treatment modulation” closed-loop system, which not only offers a novel approach to validate the targeting specificity of VEGF inhibitors but also advances anti-angiogenic therapy from empirical combinations to spatiotemporal regulation. This marks the dawn of a new era in precision kinetic intervention for anti-angiogenic therapy in HNC (113).

#### 3.2.2 Prognostic targeting tests

The secretion of pro-angiogenic factors by tumor cells leads to the formation of abnormal vascular networks, which affects tumor perfusion and in turn creates a hypoxic environment that enhances tumor cell invasiveness and reduces the tumor-killing effect of immune cells. Abnormal tumor perfusion also reduces the efficacy of chemotherapy and radiotherapy. Therefore, vascular normalization has emerged as a new therapeutic strategy, and treatments that inhibit neoangiogenesis by interfering with signal transduction pathways that regulate angiogenesis and growth or by directly targeting tumor-associated endothelial cells have been proposed as promising strategies (114). In many studies, tissue expression levels of angiogenic factors have been correlated with the likelihood of tumor spread; therefore, they are considered predictive indicators for identifying patients at high risk of poor prognosis. The use of ANG2 as a biomarker helps in the diagnosis and therapeutic monitoring of Kasibo sarcoma. By measuring ANG2 levels in serum, physicians can assess a patient’s disease status and response to treatment. High levels of ANG2 are strongly associated with the presence of Kassipo sarcoma and Kassipo lymphangioma, and can therefore be used as one of the indicators for the diagnosis of these diseases. In addition, changes in ANG2 levels can be used to monitor a patient’s response to treatment. If treatment is effective, ANG2 levels may decrease; conversely, if treatment is ineffective or the disease progresses, ANG2 levels may remain elevated (115). Disease progression disease-free survival is significantly shorter in CLL patients with high expression of ANG2 (median 21 months) and significantly longer in patients expressing low levels of ANG2 (median 146 months). In addition, patients with high ANG2 expression typically have a more advanced clinical stage, a higher proportion of CD38-positive cells, an unmutated immunoglobulin status, and unfavorable cytogenetics, all of which support the involvement of ANG2 in the disease mechanism of CLL, and therefore, the expression level of ANG2 may serve as a poor predictor of disease prognosis (116).

Angiogenic treatment with bevacizumab not only enhanced the binding of ANG1 to Tie-2, but also triggered a significant decrease in circulating Tie-2 levels. It is worth mentioning that Tie-2, as a promising prognostic biomarker, is regarded as an early warning signal for disease recurrence if the increase in its circulating levels exceeds 50%, showing its promise in predicting disease progression (99, 117). The researchers’ findings are also relevant for osteosarcoma, which is a cancerous tumor. In the case of osteosarcoma, researchers have found that inhibiting VEGF gene expression using ribonucleic acid disruption significantly inhibits tumor cell proliferation and promotes apoptosis. Meanwhile, targeted therapeutic strategies against VEGF are being actively investigated in the clinic for osteosarcoma. By monitoring the expression levels of VEGF and ANG2 in the serum of osteosarcoma patients, we are able to more accurately assess the biological characteristics of the disease and provide an important reference for the adjustment of treatment plans. This approach is of great significance for improving the clinical outcome and prognosis of osteosarcoma (118, 119). The results of this approach are very important for improving the clinical outcome and prognosis of osteosarcoma (103, 104). In contrast, there was no significant difference in angiopoietin expression between basal-like cell carcinoma and squamous cell carcinoma in the peripheral vessels of basal-like cell carcinoma and squamous cell carcinoma in the head and neck region. In cancer cells, angiopoietin expression was lower in basal-like cell carcinoma and higher in squamous cell carcinoma. In addition, angiopoietin expression in basal-like cell carcinoma was associated with the stage of the tumor, whereas angiopoietin expression in squamous cell carcinoma was not associated with the stage of the tumor. These results suggest that basal-like cell carcinoma and squamous cell carcinoma differ in their angiopoietin expression patterns, which may have implications for the prognosis of both types of head and neck tumors (120).

#### 3.2.3 Targeting roles played by ANG receptors

What’s more, transient receptor potential vanilloid-like protein 4 (TRPV4) was found to be expressed at enhanced protein levels in oral squamous cell carcinoma lesion tissues, with significant differences compared to normal tissues (121). mRNA levels of TRPV4 were also upregulated and expressed in animals treated with GSK1016790A(TRPV4 agonist) (122). VEGF immunoassays showed a significant increase in protein concentration, while cisplatin or its combination with TRPV4 agonist reduced VEGF levels. These results suggest that TRPV4 agonist combined with cisplatin may modulate angiogenesis in oral squamous cell carcinoma via the Angiopoietin/Tie pathway. Moreover, cisplatin alone or with TRPV4 agonists reduced tumor volume and malignant lesion incidence. Vascular normalization, facilitated by modulating the ANG1/Tie-2 pathway, may restore structural and functional abnormalities, improving the tumor microenvironment (123). In turn, the interaction between Tie-2 and ANG1 may also enhance the intercellular and cell-extracellular matrix adhesion capacity of OSCC cells, thereby promoting tumor metastasis and invasion. Meanwhile, Tie-2 and ANG1 play an important role in cancer metastasis and may be potential biomarkers and therapeutic targets for OSCC metastasis (124). Even ANG1-mediated vascular lymphangiogenesis can be inhibited by vasopressors (125). The researchers also found that ANG is a potential biomarker and therapeutic target for OSCC metastasis (107). Meanwhile, researchers have also found that the combination of ANG with immune checkpoint inhibitors (e.g., anti-PD-L1 antibodies) can also be used for anti-tumor therapy (126). This combination therapeutic strategy can inhibit tumor growth and metastasis and activate the host immune response, thereby increasing the anti-tumor killing capacity. At the same time, this combination therapeutic strategy can also improve the immunosuppressive state in the tumor microenvironment and enhance the anti-tumor killing capacity of infiltrating cytotoxic T cells (127).

In summary, the ANG family plays a critical role in anti-angiogenic strategies for head and neck tumors, with ANG2 inhibition demonstrating significant therapeutic potential. Also, the correlation of TRPV4 with angiogenesis and tumor metastasis provides new potential biomarkers and therapeutic targets for head and neck tumor therapy. The important concept of anti-angiogenesis is significant in head and neck tumor therapy, especially for molecules such as ANG, which provide new therapeutic ideas and potential targets for the regulation of tumor angiogenesis and metastasis mechanisms.

## 4 Challenges and prospects

Elevated expression of angiopoietin in tumor tissues of different origins and in patients’ serum shows that it is closely related to tumor development and progression (128). Studies have revealed that blood angiopoietin levels correlate with the degree of malignancy of tumors, patient prognosis and other factors, and can be used as a broad-spectrum biomarker of malignant tumors for clinical practice such as tumor diagnosis and prognosis assessment (129). The Targeted anti-angiogenic therapy seems to have become an emerging strategy for the treatment of malignant tumors, but there is a lack of targeted factors that can effectively target anti-angiogenic therapy for OSCC (130). The VEGF pathway has been shown to be effective in the treatment of OSCC (111). Although blocking the VEGF pathway inhibits tumor angiogenesis, tumor cells have an escape mechanism. When the VEGF pathway is blocked, tumors use other bypass pathways to promote angiogenesis. Complex signaling pathway interactions allow other signals to drive angiogenesis even when the VEGF receptor is inhibited. Therefore, blocking VEGF alone may be insufficient and multiple strategies need to be thoroughly investigated to counteract tumor escape (105, 131). Indeed, the high level of resistance to targeted VEGF drugs, as evidenced by disease progression after initial treatment, limits their effectiveness in cancer therapy. Therefore, improved therapeutic modalities, such as combinations with other targeted agents, are needed. Although the abnormal expression of single-class ANG have been shown to play an important role in promoting tumor angiogenesis and accelerating tumor progression, the study of clusters of several classes of ANG and their ligands and receptors together on the mechanism of tumor action is still waiting to be discovered by scholars (132, 133).The role of anti-tumor angiogenesis in cardiovascular disease The effect of anti-tumor angiogenesis on blood pressure in patients with cardiovascular disease needs to be studied. The higher than normal expression of angiopoietin in the serum of patients with oral leukoplakia provides a basis for the study of the mechanism of carcinogenesis and the screening of gene probes, and helps to control some precancerous lesions at an early stage (134). Given the key role of the ANG family in angiogenesis and tumor metastasis, it is expected that ANG-targeted drugs with low side-effects will be developed in the future to achieve precision treatment of oral tumors, enhance therapeutic efficacy, reduce recurrence and metastasis, and improve the quality of life of patients. At the same time, we will improve the research model of oral tumors to simulate their growth and metastasis, which will provide a reliable basis for drug development and treatment strategies. Interdisciplinary cooperation is a development trend, combining dentistry, immunology, molecular biology and other fields to jointly promote oral tumor research and address global health challenges.

## 5 Conclusion

This article provides insight into the important role of the ANG family in head and neck tumor generation and progression. Head and neck cancer, as a common cancer worldwide, often has an unsatisfactory prognosis due to tumor heterogeneity, drug resistance and immunosuppression. In recent years, with the deepening of medical research, the close relationship between tumor development and angiogenesis has been gradually recognized, and the ANG family, as key angiogenic factors, plays a pivotal role in tumor angiogenesis. This family consists of several members, including ANG1, ANG2, ANG3 and ANG4, which promote angiogenesis by activating various signaling pathways, such as MAPK, RAS, RAP1, and PI3-AKT, which in turn promote cell growth, differentiation and proliferation. This process is critical for tumor growth and metastasis. Notably, although VEGF has been mainly used as the main target for the treatment of aberrant blood vessel growth in the past, the ANG-TIE-2 pathway has also gradually shown its potential in anti-tumor induced angiogenesis. This finding provides new ideas and approaches for the treatment of head and neck tumors. An in-depth understanding of the relationship between the ANG family and head and neck tumors will not only help us to gain a more comprehensive understanding of the mechanisms of tumor generation and development, but is also expected to provide more precise and effective strategies for the treatment of head and neck tumors. Through in-depth study of the mechanism of action of the ANG family, we may be able to develop new therapeutic approaches to improve patient outcomes and quality of life. Therefore, future studies should further focus on the value of the ANG family in the treatment of head and neck tumors and explore its potential in anti-tumor therapy, with a view to bringing better therapeutic effects and prognosis to patients. Meanwhile, the complex network relationship between ANG family and other growth factors and cytokines in order to reveal their specific mechanism of action in the tumor microenvironment.

## References

[1] Leonel AC Bonan RF Pinto MB Kowalski LP Perez DE. The pesticides use and the risk for head and neck cancer: A review of case-control studies. Med Oral Patol Oral Cir Bucal. (2021) 26:e56–63. doi: 10.4317/medoral.23962 PMC7806356 32701932

[2] Jumaniyazova E Lokhonina A Dzhalilova D Kosyreva A Fatkhudinov T. Role of microenvironmental components in head and neck squamous cell carcinoma. J Pers Med. (2023) 13(11):1616. doi: 10.3390/jpm13111616 38003931 PMC10672525

[3] Johnson DE Burtness B Leemans CR Lui VWY Bauman JE Grandis JR. Head and neck squamous cell carcinoma. Nat Rev Dis Primers. (2020) 6(1):92. doi: 10.1038/s41572-020-00224-3 33243986 PMC7944998

[4] da Costa Sousa MG Vignolo SM Franca CM Mereness J Alves Fraga MA Silva-Sousa AC. Engineering models of head and neck and oral cancers on-a-chip. Biomicrofluidics. (2024) 18(2):021502. doi: 10.1063/5.0186722 38464668 PMC10919958

[5] Mannan A Dhiamn S Garg N Singh TG. Pharmacological modulation of Sonic Hedgehog signaling pathways in Angiogenesis: A mechanistic perspective. Dev Biol. (2023) 504:58–74. doi: 10.1016/j.ydbio.2023.09.009 37739118

[6] Shibuya M. Vascular endothelial growth factor (VEGF) and its receptor (VEGFR) signaling in angiogenesis: A crucial target for anti- and pro-angiogenic therapies. Genes Cancer. (2011) 2:1097–105. doi: 10.1177/1947601911423031 PMC3411125 22866201

[7] Thapa K Khan H Kaur G Kumar P Singh TG. Therapeutic targeting of angiopoietins in tumor angiogenesis and cancer development. Biochem Biophys Res Commun. (2023) 687:149130. doi: 10.1016/j.bbrc.2023.149130 37944468

[8] Durante S Dunet V Gorostidi F Mitsakis P Schaefer N Delage J. Head and neck tumors angiogenesis imaging with (68)Ga-NODAGA-RGD in comparison to (18)F-FDG PET/CT: a pilot study. EJNMMI Res. (2020) 10:47. doi: 10.1186/s13550-020-00638-w 32382869 PMC7205972

[9] Hamilton JL Nagao M Levine BR Chen D Olsen BR Im H-J. Targeting VEGF and its receptors for the treatment of osteoarthritis and associated pain. J Bone Mineral Res. (2016) 31:911–24. doi: 10.1002/jbmr.2828 PMC4863467 27163679

[10] Yu X Ye F. Role of angiopoietins in development of cancer and neoplasia associated with viral infection. Cells. (2020) 9(2):457. doi: 10.3390/cells9020457 32085414 PMC7072744

[11] Khan KA Wu FTH Cruz-Munoz W Kerbel RS. Ang2 inhibitors and Tie2 activators: potential therapeutics in perioperative treatment of early stage cancer. EMBO Mol Med. (2021) 13(7):e08253. doi: 10.15252/emmm.201708253 34125494 PMC8261516

[12] Gheblawi M Wang K Viveiros A Nguyen Q Zhong J-C Turner AJ. Angiotensin-converting enzyme 2: SARS-coV-2 receptor and regulator of the renin-angiotensin system. Circ Res. (2020) 126:1456–74. doi: 10.1161/CIRCRESAHA.120.317015 PMC7188049 32264791

[13] Zhu C Gu L Liu Z Li J Yao M Fang C. Correlation between vascular endothelial growth factor pathway and immune microenvironment in head and neck squamous cell carcinoma. BMC Cancer. (2021) 21:836. doi: 10.1186/s12885-021-08547-4 34284746 PMC8290614

[14] Thurston G Rudge JS Ioffe E Zhou H Ross L Croll SD. Angiopoietin-1 protects the adult vasculature against plasma leakage. Nat America Inc. (2000) 6(4):460–3. doi: 10.1038/74725 10742156

[15] Park JH Choi H Kim YB Kim YS Sheen SS Choi J-H. Serum angiopoietin-1 as a prognostic marker in resected early stage lung cancer. Lung Cancer. (2009) 66:359–64. doi: 10.1016/j.lungcan.2009.03.002 19339077

[16] Atesoglu EB Tarkun P Mehtap O Demirsoy ET Atalay F Maden M. Serum Angiopoietin Levels are Different in Acute and Chronic Myeloid Neoplasms: Angiopoietins do not only Regulate Tumor Angiogenesis. Indian J Hematol Blood Transfusion. (2015) 32:162–7. doi: 10.1007/s12288-015-0548-8 PMC4789009 27065577

[17] David S Ghosh CC Mukherjee A Parikh SM. Angiopoietin-1 requires IQ domain GTPase-activating protein 1 to activate rac1 and promote endothelial barrier defense. Arteriosclerosis Thrombosis Vasc Biol. (2011) 31:2643–52. doi: 10.1161/ATVBAHA.111.233189 PMC3249617 21885850

[18] Fukuhara S Sako K Minami T Noda K Kim HZ Kodama T. Differential function of Tie2 at cell–cell contacts and cell–substratum contacts regulated by angiopoietin-1. Nat Cell Biol. (2008) 10:513–26. doi: 10.1038/ncb1714 18425120

[19] Suri C Jones PF Patan S Bartunkova S Maisonpierre PC Davis S. Requisite role of angiopoietin-1, a ligand for the TIE2 receptor, during embryonic angiogenesis. Cell. (1996) 87(7):1171–80. doi: 10.1016/S0092-8674(00)81813-9 8980224

[20] Yu X Seegar TCM Dalton AC Tzvetkova-Robev D Goldgur Y Rajashankar KR. Structural basis for angiopoietin-1–mediated signaling initiation. Proc Natl Acad Sci. (2013) 110:7205–10. doi: 10.1073/pnas.1216890110 PMC3645508 23592718

[21] Donnem T Hu J Ferguson M Adighibe O Snell C Harris AL. Vessel co-option in primary human tumors and metastases: an obstacle to effective anti-angiogenic treatment? Cancer Med. (2013) 2:427–36. doi: 10.1002/cam4.2013.2.issue-4 PMC3799277 24156015

[22] Michael IP Orebrand M Lima M Pereira B Volpert O Quaggin SE. Angiopoietin-1 deficiency increases tumor metastasis in mice. BMC Cancer. (2017) 17(1):539. doi: 10.1186/s12885-017-3531-y 28800750 PMC5553747

[23] Fiedler U Scharpfenecker M Koidl S Hegen A Grunow V Schmidt JM. The Tie-2 ligand Angiopoietin-2 is stored in and rapidly released upon stimulation from endothelial cell Weibel-Palade bodies. Blood. (2004) 103:4150–6. doi: 10.1182/blood-2003-10-3685 14976056

[24] Hakanpaa L Sipila T Leppanen V-M Gautam P Nurmi H Jacquemet G. Endothelial destabilization by angiopoietin-2 via integrin β1 activation. Nat Commun. (2015) 6:5962. doi: 10.1038/ncomms6962 25635707 PMC4316742

[25] Avraamides CJ Garmy-Susini B Varner JA. Integrins in angiogenesis and lymphangiogenesis. Nat Rev Cancer. (2008) 8:604–17. doi: 10.1038/nrc2353 PMC2577722 18497750

[26] Mezu-Ndubuisi OJ Maheshwari A. The role of integrins in inflammation and angiogenesis. Pediatr Res. (2020) 89:1619–26. doi: 10.1038/s41390-020-01177-9 PMC8249239 33027803

[27] Davis S Aldrich TH Jones PF Acheson A Compton DL Jain V. Isolation of angiopoietin-1, a ligand for the TIE2 receptor, by secretion-trap expression cloning. Cell. (1996) 87(7):1161–9. doi: 10.1016/S0092-8674(00)81812-7 8980223

[28] Davis S Papadopoulos N Aldrich TH Maisonpierre PC Huang T Kovac L. Angiopoietins have distinct modular domains essential for receptor binding, dimerization and superclustering. Nat Struct Biol. (2002) 10:38–44. doi: 10.1038/nsb880 12469114

[29] Komici K Faris P Negri S Rosti V García-Carrasco M Mendoza-Pinto C. Systemic lupus erythematosus, endothelial progenitor cells and intracellular Ca2+ signaling: A novel approach for an old disease. J Autoimmun. (2020) 112:102486. doi: 10.1016/j.jaut.2020.102486 32482487

[30] Blecharz KG Frey D Schenkel T Prinz V Bedini G Krug SM. Autocrine release of angiopoietin-2 mediates cerebrovascular disintegration in Moyamoya disease. J Cereb Blood Flow Metab. (2016) 37:1527–39. doi: 10.1177/0271678X16658301 PMC5453470 27381827

[31] Ferrara N Hillan KJ Gerber H-P Novotny W. Discovery and development of bevacizumab, an anti-VEGF antibody for treating cancer. Nat Rev Drug Discovery. (2004) 3:391–400. doi: 10.1038/nrd1381 15136787

[32] Lahiry P Torkamani A Schork NJ Hegele RA. Kinase mutations in human disease: interpreting genotype–phenotype relationships. Nat Rev Genet. (2010) 11:60–74. doi: 10.1038/nrg2707 20019687

[33] Ghosh S Sullivan CAW Zerkowski MP Molinaro AM Rimm DL Camp RL. High levels of vascular endothelial growth factor and its receptors (VEGFR-1, VEGFR-2, neuropilin-1) are associated with worse outcome in breast cancer. Hum Pathology. (2008) 39:1835–43. doi: 10.1016/j.humpath.2008.06.004 PMC2632946 18715621

[34] Zarychta E Bielawski K Wrzeszcz K Rhone P Ruszkowska-Ciastek B. Unraveling the Angiogenic Puzzle: Pre-Treatment sVEGFR1 and sVEGFR2 Levels as Promising Prognostic Indicators in Early-Stage Breast Cancer Patients. Int J Mol Sci. (2023) 24(17):13508. doi: 10.3390/ijms241713508 37686312 PMC10487545

[35] Akwii RG Sajib MS Zahra FT Mikelis CM. Role of angiopoietin-2 in vascular physiology and pathophysiology. Cells. (2019) 8(5):471. doi: 10.3390/cells8050471 31108880 PMC6562915

[36] Shimoda H Bernas MJ Witte MH Gale NW Yancopoulos GD Kato S. Abnormal recruitment of periendothelial cells to lymphatic capillaries in digestive organs of angiopoietin-2-deficient mice. Cell Tissue Res. (2007) 328:329–37. doi: 10.1007/s00441-006-0360-8 17235601

[37] Lee HJ Cho C-H Hwang S-J Choi H-H Kim K-T Ahn SY. Biological characterization of angiopoietin-3 and angiopoietin-4. FASEB J. (2004) 18:1200–8. doi: 10.1096/fj.03-1466com 15284220

[38] Xu Y Liu Y-j Yu Q. Angiopoietin-3 inhibits pulmonary metastasis by inhibiting tumor angiogenesis. Cancer Res. (2004) 64:6119–26. doi: 10.1158/0008-5472.CAN-04-1054 15342395

[39] Koritzinsky M Andersen S Donnem T Al-Shibli K Al-Saad S Stenvold H. Prognostic impacts of angiopoietins in NSCLC tumor cells and stroma: VEGF-A impact is strongly associated with ang-2. PloS One. (2011) 6(5):e19773. doi: 10.1371/journal.pone.0019773 21603628 PMC3095634

[40] Olsen Mwb D Ley C Junker N Hansen AJ Lund EL Kristjansen PEG. Angiopoietin-4 inhibits angiogenesis and reduces interstitial fluid pressure. Neoplasia. (2006) 8:364–72. doi: 10.1593/neo.06127 PMC1592453 16790085

[41] Cascone I Napione L Maniero F Serini G Bussolino F. Stable interaction between α5β1 integrin and Tie2 tyrosine kinase receptor regulates endothelial cell response to Ang-1. J Cell Biol. (2005) 170:993–1004. doi: 10.1083/jcb.200507082 16157706 PMC2171441

[42] Dalton AC Shlamkovitch T Papo N Barton WA. Correction: constitutive association of tie1 and tie2 with endothelial integrins is functionally modulated by angiopoietin-1 and fibronectin. PloS One. (2017) 12(5):e0179059. doi: 10.1371/journal.pone.0179059 28562653 PMC5451130

[43] Giannotta M Trani M Dejana E. VE-cadherin and endothelial adherens junctions: active guardians of vascular integrity. Dev Cell. (2013) 26:441–54. doi: 10.1016/j.devcel.2013.08.020 24044891

[44] Cossutta M Darche M Carpentier G Houppe C Ponzo M Raineri F. Weibel-palade bodies orchestrate pericytes during angiogenesis. Arteriosclerosis Thrombosis Vasc Biol. (2019) 39:1843–58. doi: 10.1161/ATVBAHA.119.313021 31315435

[45] Koh GY. Orchestral actions of angiopoietin-1 in vascular regeneration. Trends Mol Med. (2013) 19:31–9. doi: 10.1016/j.molmed.2012.10.010 23182855

[46] Injune Kim HGK So J-N Kim JH Kwak HJ Koh GY. Angiopoietin-1 regulates endothelial cell survival through the phosphatidylinositol 3-Kinase-Akt signal transduction pathway. Circ Res. (2000) 86:24–9. doi: 10.1161/01.res.86.1.24 10625301

[47] Wilhelm K Happel K Eelen G Schoors S Oellerich MF Lim R. FOXO1 couples metabolic activity and growth state in the vascular endothelium. Nature. (2016) 529:216–20. doi: 10.1038/nature16498 PMC5380221 26735015

[48] Potente M Urbich C Sasaki K-i Hofmann WK Heeschen C Aicher A. Involvement of Foxo transcription factors in angiogenesis and postnatal neovascularization. J Clin Invest. (2005) 115:2382–92. doi: 10.1172/JCI23126 PMC1184037 16100571

[49] Daly C Pasnikowski E Burova E Wong V Aldrich TH Griffiths J. Angiopoietin-2 functions as an autocrine protective factor in stressed endothelial cells. ProcNatl Acad Sci USA. (2006) 103:15491–6. doi: 10.1073/pnas.0607538103 PMC1592534 17030814

[50] Kamoun S Wróblewski T Spiridon L Martin EC Petrescu A-J Cavanaugh K. Genome-wide functional analyses of plant coiled–coil NLR-type pathogen receptors reveal essential roles of their N-terminal domain in oligomerization, networking, and immunity. PloS Biol. (2018) 16(12):e2005821. doi: 10.1371/journal.pbio.2005821 30540748 PMC6312357

[51] Leppänen V-M Saharinen P Alitalo K. Structural basis of Tie2 activation and Tie2/Tie1 heterodimerization. Proc Natl Acad Sci. (2017) 114:4376–81. doi: 10.1073/pnas.1616166114 PMC5410806 28396439

[52] Saharinen P Eklund L Miettinen J Wirkkala R Anisimov A Winderlich M. Angiopoietins assemble distinct Tie2 signalling complexes in endothelial cell–cell and cell–matrix contacts. Nat Cell Biol. (2008) 10:527–37. doi: 10.1038/ncb1715 18425119

[53] Nie X Fei W Zhou H Li M Gao M. Research progress on the relationship between the Ang-Tie2 pathway and angiogenesis. Pract J Clin Med. (2022) 02:203–6. doi: 10.3969/j.issn

[54] De Palma M Biziato D Petrova TV. Microenvironmental regulation of tumour angiogenesis. Nat Rev Cancer. (2017) 17:457–74. doi: 10.1038/nrc.2017.51 28706266

[55] Dong Z Chen J Yang X Zheng W Wang L Fang M. Ang-2 promotes lung cancer metastasis by increasing epithelial-mesenchymal transition. Oncotarget. (2018) 9:12705–17. doi: 10.18632/oncotarget.24061 PMC5849167 29560103

[56] Marla V Hegde V Shrestha A. Relationship of angiogenesis and oral squamous cell carcinoma. Kathmandu Univ Med J (KUMJ). (2015) 13:178–85. doi: 10.3126/kumj.v13i2.16796 26643840

[57] Paul M Poyan Mehr A Kreutz R. Physiology of local renin-angiotensin systems. Physiol Rev. (2006) 86:747–803. doi: 10.1152/physrev.00036.2005 16816138

[58] Lisabeth EM Falivelli G Pasquale EB. Eph receptor signaling and ephrins. Cold Spring Harbor Perspect Biol. (2013) 5:a009159–a. doi: 10.1101/cshperspect.a009159 PMC3753714 24003208

[59] Atanasov G Dino K Schierle K Dietel C Aust G Pratschke J. Recipient hepatic tumor-associated immunologic infiltrates predict outcomes after liver transplantation for hepatocellular carcinoma. Ann Transplantation. (2020) 25:e919414. doi: 10.12659/AOT.919414 PMC7092657 32165607

[60] Falcon BL Chintharlapalli S Uhlik MT Pytowski B. Antagonist antibodies to vascular endothelial growth factor receptor 2 (VEGFR-2) as anti-angiogenic agents. Pharmacol Ther. (2016) 164:204–25. doi: 10.1016/j.pharmthera.2016.06.001 27288725

[61] Mazzieri R Pucci F Moi D Zonari E Ranghetti A Berti A. Targeting the ANG2/TIE2 axis inhibits tumor growth and metastasis by impairing angiogenesis and disabling rebounds of proangiogenic myeloid cells. Cancer Cell. (2011) 19:512–26. doi: 10.1016/j.ccr.2011.02.005 21481792

[62] Hosaka K Andersson P Wu J He X Du Q Jing X. KRAS mutation-driven angiopoietin 2 bestows anti-VEGF resistance in epithelial carcinomas. ProcNatl Acad Sci USA. (2023) 120:e2303740120. doi: 10.1073/pnas.2303740120 PMC10629547 37428914

[63] Kim M Allen B Korhonen EA Nitschké M Yang HW Baluk P. Opposing actions of angiopoietin-2 on Tie2 signaling and FOXO1 activation. J Clin Invest. (2016) 126:3511–25. doi: 10.1172/JCI84871 PMC5004955 27548529

[64] Nasarre P Thomas M Kruse K Helfrich I Wolter V Deppermann C. Host-derived angiopoietin-2 affects early stages of tumor development and vessel maturation but is dispensable for later stages of tumor growth. Cancer Res. (2009) 69:1324–33. doi: 10.1158/0008-5472.CAN-08-3030 PMC3514474 19208839

[65] Abdul Pari AA Singhal M Hübers C Mogler C Schieb B Gampp A. Tumor cell–derived angiopoietin-2 promotes metastasis in melanoma. Cancer Res. (2020) 80:2586–98. doi: 10.1158/0008-5472.CAN-19-2660 PMC7611175 32303578

[66] Shim WSN Ho IAW Wong PEH. Angiopoietin: A TIE(d) balance in tumor angiogenesis. Mol Cancer Res. (2007) 5:655–65. doi: 10.1158/1541-7786.MCR-07-0072 17634421

[67] Holopainen T Saharinen P D’Amico G Lampinen A Eklund L Sormunen R. Effects of angiopoietin-2-blocking antibody on endothelial cell–cell junctions and lung metastasis. JNCI: J Natl Cancer Institute. (2012) 104:461–75. doi: 10.1093/jnci/djs009 PMC3309130 22343031

[68] ten Voorde AMW Wierenga APA Nell RJ van der Velden PA Luyten GPM Verdijk RM. In uveal melanoma, angiopoietin-2 but not angiopoietin-1 is increased in high-risk tumors, providing a potential druggable target. Cancers. (2021) 13(16):3986. doi: 10.3390/cancers13163986 34439141 PMC8391938

[69] Ribatti D Tamma R Annese T. Epithelial-mesenchymal transition in cancer: A historical overview. Transl Oncol. (2020) 13:100773. doi: 10.1016/j.tranon.2020.100773 32334405 PMC7182759

[70] Farahzadi R Valipour B Fathi E Pirmoradi S Molavi O Montazersaheb S. Oxidative stress regulation and related metabolic pathways in epithelial-mesenchymal transition of breast cancer stem cells. Stem Cell Res Ther. (2023) 14:342. doi: 10.1186/s13287-023-03571-6 38017510 PMC10685711

[71] Alqurashi YE Al-Hetty H Ramaiah P Fazaa AH Jalil AT Alsaikhan F. Harnessing function of EMT in hepatocellular carcinoma: From biological view to nanotechnological standpoint. Environ Res. (2023) 227:115683. doi: 10.1016/j.envres.2023.115683 36933639

[72] Fagiani E Christofori G. Angiopoietins in angiogenesis. Cancer Letters. (2013) 328:18–26. doi: 10.1016/j.canlet.2012.08.018 22922303

[73] Chanmee T Ontong P Konno K Itano N. Tumor-associated macrophages as major players in the tumor microenvironment. Cancers. (2014) 6:1670–90. doi: 10.3390/cancers6031670 PMC4190561 25125485

[74] Peng Q Li M Wang Z Jiang M Yan X Lei S. Polarization of tumor-associated macrophage is associated with tumor vascular normalization by endostatin. Thorac Cancer. (2013) 4:295–305. doi: 10.1111/tca.2013.4.issue-3 28920238

[75] De Palma M Naldini L. Tie2-expressing monocytes (TEMs): Novel targets and vehicles of anticancer therapy? Biochim Biophys Acta (BBA) - Rev Cancer. (2009) 1796:5–10. doi: 10.1016/j.bbcan.2009.04.001 19362584

[76] Argiris A Karamouzis MV Raben D Ferris RL. Head and neck cancer. Lancet. (2008) 371:1695–709. doi: 10.1016/S0140-6736(08)60728-X PMC7720415 18486742

[77] Zhang Z Shi B Zhang C eds. Oral and maxillofacial surgery. 8th ed. Beijing: Science Press (2020). 423 p.

[78] Zhang Y Weinberg RA. Epithelial-to-mesenchymal transition in cancer: complexity and opportunities. Front Med. (2018) 12:361–73. doi: 10.1007/s11684-018-0656-6 PMC6186394 30043221

[79] Jeong IS Roh JL Cho KJ Choi SH Nam SY Kim SY. Risk factors for survival and distant metastasis in 125 patients with head and neck adenoid cystic carcinoma undergoing primary surgery. J Cancer Res Clin Oncol. (2020) 146:1343–50. doi: 10.1007/s00432-020-03170-5 PMC11804535 32144535

[80] Liang B Xu H. Analysis of risk factors for postoperative cognitive dysfunction in oral cancer patients. Chin J Oral Maxillofac Surg. (2015) 04:352–5. doi: 10.1002/cam4.3428

[81] Liu S Yang J Lu H Wu Y Yang W Xu W. Adenoid cystic carcinoma of submandibular gland: Emphasis on locoregional metastasis and prognosis. Oral Dis. (2024) 30:1152–62. doi: 10.1111/odi.14478 36564993

[82] Liu M Xun L. Analysis of factors related to postoperative respiratory tract infections in oral and maxillofacial tumor patients. J Nurs PLA. (2003) 09:21–3. doi: 10.11816/cn.ni.2015-134152

[83] Li C. Application of psychological nursing intervention in elderly patients after oral tumor surgery. J Qilu Nurs. (2017) 20:116–7. doi: 10.3969/j.issn.1006-7256.2017.20.053

[84] Folkman J. Fundamental concepts of the angiogenic process. Curr Mol Med. (2003) 3:643–51. doi: 10.2174/1566524033479465 14601638

[85] Kerbel RS Shaked Y. The potential clinical promise of ‘multimodality’ metronomic chemotherapy revealed by preclinical studies of metastatic disease. Cancer Letters. (2017) 400:293–304. doi: 10.1016/j.canlet.2017.02.005 28202353

[86] Jo G Bae J Hong HJ Han A-r Kim D-K Hong SP. Structural insights into the clustering and activation of Tie2 receptor mediated by Tie2 agonistic antibody. Nat Commun. (2021) 12(1):6287. doi: 10.1038/s41467-021-26620-1 34725372 PMC8560823

[87] Lilhore UK Poongodi M Kaur A Simaiya S Algarni AD Elmannai H. Hybrid model for detection of cervical cancer using causal analysis and machine learning techniques. Comput Math Methods Med. (2022) 2022:1–17. doi: 10.1155/2022/4688327 PMC9095387 35572826

[88] Gengenbacher N Singhal M Augustin HG. Preclinical mouse solid tumour models: status quo, challenges and perspectives. Nat Rev Cancer. (2017) 17:751–65. doi: 10.1038/nrc.2017.92 29077691

[89] Marzo T Ferraro G Cucci LM Pratesi A Hansson Ö Satriano C. Oxaliplatin inhibits angiogenin proliferative and cell migration effects in prostate cancer cells. J Inorg Biochem. (2022) 226:111657. doi: 10.1016/j.jinorgbio.2021.111657 34784565

[90] Xiao Y Duan Y Wang Y Yin X. Resveratrol suppresses Malignant progression of oral squamous cell carcinoma cells by inducing the ZNF750/RAC1 signaling pathway. Bioengineered. (2021) 12:2863–73. doi: 10.1080/21655979.2021.1940616 PMC8806518 34176441

[91] Oon CE Subramaniam AV Ooi LY Yehya AHS Lee YT Kaur G. BZD9L1 benzimidazole analogue hampers colorectal tumor progression by impeding angiogenesis. World J Gastrointestinal Oncol. (2023) 15:810–27. doi: 10.4251/wjgo.v15.i5.810 PMC10237024 37275453

[92] Yoshioka N Wang L Kishimoto K Tsuji T Hu GF. A therapeutic target for prostate cancer based on angiogenin-stimulated angiogenesis and cancer cell proliferation. ProcNatl Acad Sci USA. (2006) 103:14519–24. doi: 10.1073/pnas.0606708103 PMC1599992 16971483

[93] Tsuji T Sun Y Kishimoto K Olson KA Liu S Hirukawa S. Angiogenin is translocated to the nucleus of heLa cells and is involved in ribosomal RNA transcription and cell proliferation. Cancer Res. (2005) 65:1352–60. doi: 10.1158/0008-5472.CAN-04-2058 15735021

[94] Vassilakopoulou M Psyrri A Argiris A. Targeting angiogenesis in head and neck cancer. Oral Oncol. (2015) 51:409–15. doi: 10.1016/j.oraloncology.2015.01.006 25680863

[95] Augustin HG Young Koh G Thurston G Alitalo K. Control of vascular morphogenesis and homeostasis through the angiopoietin–Tie system. Nat Rev Mol Cell Biol. (2009) 10:165–77. doi: 10.1038/nrm2639 19234476

[96] Gerald D Chintharlapalli S Augustin HG Benjamin LE. Angiopoietin-2: an attractive target for improved antiangiogenic tumor therapy. Cancer Res. (2013) 73:1649–57. doi: 10.1158/0008-5472.CAN-12-4697 23467610

[97] Kishimoto K Yoshida S Ibaragi S Yoshioka N Okui T Hu G-f. Hypoxia-induced up-regulation of angiogenin, besides VEGF, is related to progression of oral cancer. Oral Oncol. (2012) 48:1120–7. doi: 10.1016/j.oraloncology.2012.05.009 22694909

[98] Park J-S Kim I-K Han S Park I Kim C Bae J. Normalization of tumor vessels by tie2 activation and ang2 inhibition enhances drug delivery and produces a favorable tumor microenvironment. Cancer Cell. (2017) 31:157–8. doi: 10.1016/j.ccell.2016.12.009 28073001

[99] Piao Y Park SY Henry V Smith BD Tiao N Flynn DL. Novel MET/TIE2/VEGFR2 inhibitor altiratinib inhibits tumor growth and invasiveness in bevacizumab-resistant glioblastoma mouse models. Neuro-Oncology. (2016) 18:1230–41. doi: 10.1093/neuonc/now030 PMC4998992 26965451

[100] Chen L Hu G-f. Angiogenin-mediated ribosomal RNA transcription as a molecular target for treatment of head and neck squamous cell carcinoma. Oral Oncol. (2010) 46:648–53. doi: 10.1016/j.oraloncology.2010.06.011 PMC2932836 20656548

[101] Dowlati A Vlahovic G Natale RB Rasmussen E Singh I Hwang YC. First-in-human study of AMG 780, an angiopoietin-1 and -2 inhibitor, in patients with advanced solid tumors. Clin Cancer Res. (2016) 22:4574–84. doi: 10.1158/1078-0432.CCR-15-2145 27076631

[102] Heery CR O’Sullivan-Coyne G Madan RA Cordes L Rajan A Rauckhorst M. Avelumab for metastatic or locally advanced previously treated solid tumours (JAVELIN Solid Tumor): a phase 1a, multicohort, dose-escalation trial. Lancet Oncol. (2017) 18:587–98. doi: 10.1016/S1470-2045(17)30239-5 PMC6387686 28373007

[103] Ferrara N. VEGF and intraocular neovascularization: from discovery to therapy. Trans Vision Sci Technol. (2016) 5(2):10. doi: 10.1167/tvst.5.2.10 PMC4790412 26981332

[104] Jayson GC Kerbel R Ellis LM Harris AL. Antiangiogenic therapy in oncology: current status and future directions. Lancet. (2016) 388:518–29. doi: 10.1016/S0140-6736(15)01088-0 26853587

[105] Saba NF Vijayvargiya P Vermorken JB Rodrigo JP Willems SM Zidar N. Targeting angiogenesis in squamous cell carcinoma of the head and neck: opportunities in the immunotherapy era. Cancers (Basel). (2022) 14(5):1202. doi: 10.3390/cancers14051202 35267508 PMC8909398

[106] Hyytiäinen A Wahbi W Väyrynen O Saarilahti K Karihtala P Salo T. Angiogenesis inhibitors for head and neck squamous cell carcinoma treatment: is there still hope? Front Oncol. (2021) 11:683570. doi: 10.3389/fonc.2021.683570 34195084 PMC8236814

[107] Monk BJ Poveda A Vergote I Raspagliesi F Fujiwara K Bae D-S. Anti-angiopoietin therapy with trebananib for recurrent ovarian cancer (TRINOVA-1): a randomised, multicentre, double-blind, placebo-controlled phase 3 trial. Lancet Oncol. (2014) 15:799–808. doi: 10.1016/S1470-2045(14)70244-X 24950985

[108] Beaussier M Delbos A Maurice-Szamburski A Ecoffey C Mercadal L. Perioperative use of intravenous lidocaine. Drugs. (2018) 78:1229–46. doi: 10.1007/s40265-018-0955-x 30117019

[109] Kishimoto K Yoshida S Ibaragi S Yoshioka N Hu GF Sasaki A. Neamine inhibits oral cancer progression by suppressing angiogenin-mediated angiogenesis and cancer cell proliferation. Anticancer Res. (2014) 34:2113–21. doi: 10.1158/1078-0432.ccr-08-2593 PMC4757496 24778013

[110] Harney AS Karagiannis GS Pignatelli J Smith BD Kadioglu E Wise SC. The selective tie2 inhibitor rebastinib blocks recruitment and function of tie2Hi macrophages in breast cancer and pancreatic neuroendocrine tumors. Mol Cancer Ther. (2017) 16:2486–501. doi: 10.1158/1535-7163.MCT-17-0241 PMC5669998 28838996

[111] Narayan V Thompson EW Demissei B Ho JE Januzzi JL Jr. Ky B. Mechanistic biomarkers informative of both cancer and cardiovascular disease: JACC state-of-the-art review. J Am Coll Cardiol. (2020) 75:2726–37. doi: 10.1016/j.jacc.2020.03.067 PMC7261288 32466889

[112] Brett VE Dignat George F James C. Circulating endothelial cells in pathophysiology. Curr Opin Hematol. (2024) 31:148–54. doi: 10.1097/MOH.0000000000000814 38362895

[113] Danova M Comolli G Manzoni M Torchio M Mazzini G. Flow cytometric analysis of circulating endothelial cells and endothelial progenitors for clinical purposes in oncology: A critical evaluation. Mol Clin Oncol. (2016) 4:909–17. doi: 10.3892/mco.2016.823 PMC4888001 27284422

[114] Viallard C Larrivée B. Tumor angiogenesis and vascular normalization: alternative therapeutic targets. Angiogenesis. (2017) 20:409–26. doi: 10.1007/s10456-017-9562-9 28660302

[115] Engel ER Le Cras TD Ricci KW. How we use angiopoietin-2 in the diagnosis and management of vascular anomalies. Pediatr Blood Cancer. (2024) 71(5):e30921. doi: 10.1002/pbc.30921 38439088 PMC12919156

[116] Maffei R Marasca R Martinelli S Castelli I Santachiara R Morandi E. Angiopoietin-2 expression in B-cell chronic lymphocytic leukemia: association with clinical outcome and immunoglobulin heavy-chain mutational status. Leukemia. (2007) 21:1312–5. doi: 10.1038/sj.leu.2404650 17361220

[117] Zhou C Clamp A Backen A Berzuini C Renehan A Banks RE. Systematic analysis of circulating soluble angiogenesis-associated proteins in ICON7 identifies Tie2 as a biomarker of vascular progression on bevacizumab. Br J Cancer. (2016) 115:228–35. doi: 10.1038/bjc.2016.194 PMC4947705 27351218

[118] Song Y. Expression and clinical significance of vascular endothelial growth factor and angiopoietin-2 in osteosarcoma detection. Chin J Public Health Eng. (2017) 03:370–1.

[119] Xing W Song F Hu W. Expression and significance of Ang-2 and VEGF in serum of osteosarcoma patients. Mod Oncol. (2015) 20:3021–3. doi: 10.3969/j.issn.1672-4992.2015.20.42

[120] Marioni G Staffieri A Savastano M Marino F Giacomelli L Lionello M. Angiogenin expression in head and neck basaloid and conventional squamous cell carcinoma: a site- and stage-matched comparison. J Oral Pathol Med. (2011) 40:55–60. doi: 10.1111/j.1600-0714.2010.00942.x 20923443

[121] Fujii S Tajiri Y Hasegawa K Matsumoto S Yoshimoto RU Wada H. The TRPV4-AKT axis promotes oral squamous cell carcinoma cell proliferation via CaMKII activation. Lab Invest. (2020) 100:311–23. doi: 10.1038/s41374-019-0357-z 31857698

[122] Sakakibara A Sakakibara S Kusumoto J Takeda D Hasegawa T Akashi M. Upregulated expression of transient receptor potential cation channel subfamily V receptors in mucosae of patients with oral squamous cell carcinoma and patients with a history of alcohol consumption or smoking. PloS One. (2017) 12:e0169723. doi: 10.1371/journal.pone.0169723 28081185 PMC5230781

[123] Yahya F Mohd Bakri M Hossain M Syed Abdul Rahman S Mohammed Alabsi A Ramanathan A. Combination treatment of TRPV4 agonist with cisplatin promotes vessel normalization in an animal model of oral squamous cell carcinoma. Medicina. (2022) 58(9):1229. doi: 10.3390/medicina58091229 36143906 PMC9504292

[124] Kitajima D Kasamatsu A Nakashima D Miyamoto I Kimura Y Endo-Sakamoto Y. Evidence for critical role of Tie2/Ang1 interaction in metastatic oral cancer. Oncol Lett. (2018) 15:7237–42. doi: 10.3892/ol.2018.8212 PMC5920920 29731883

[125] Hwang-Bo J Yoo KH Park JH Jeong HS Chung IS. Recombinant canstatin inhibits angiopoietin-1-induced angiogenesis and lymphangiogenesis. Int J Cancer. (2011) 131:298–309. doi: 10.1002/ijc.26353 21823121

[126] Su N-W Dai S-H Hsu K Chang K-M Ko C-C Kao C-W. PD-L1-positive circulating endothelial progenitor cells associated with immune response to PD-1 blockade in patients with head and neck squamous cell carcinoma. Cancer Immunology Immunotherapy. (2024) 73(1):3. doi: 10.1007/s00262-023-03595-0 38175307 PMC10992497

[127] Zhan J Zhang M Zhou L He C. Combination of immune checkpoint blockade and targeted gene regulation of angiogenesis for facilitating antitumor immunotherapy. Front Bioeng Biotechnol. (2023) 11:1065773. doi: 10.3389/fbioe.2023.1065773 36994358 PMC10040836

[128] Alessandrini L Astolfi L Daloiso A Sbaraglia M Mondello T Zanoletti E. Diagnostic, prognostic, and therapeutic role for angiogenesis markers in head and neck squamous cell carcinoma: A narrative review. Int J Mol Sci. (2023) 24(13):10733. doi: 10.3390/ijms241310733 37445908 PMC10341715

[129] Wang D Xu Z. Clinical significance of angiopoietin detection. J Pract Oncol. (2007) 04:362–6. doi: 10.3969/j.issn.1001-1692.2007.04.030

[130] Li Q Zhou Y Cai Y Li C. Research progress on the expression and function of the angiopoietin family in oral squamous cell carcinoma. J Cancer Control Treat. (2016) 03:181–5. doi: 10.3969/j.issn.1674-0904.2016.03.011

[131] Alfranca A López-Oliva JM Genís L López-Maderuelo D Mirones I Salvado D. PGE2 induces angiogenesis via MT1-MMP–mediated activation of the TGFβ/Alk5 signaling pathway. Blood. (2008) 112:1120–8. doi: 10.1182/blood-2007-09-112268 18541723

[132] Hao X Yang D Wang X Liang X. Expression of inhibitor of differentiation-1 in oral squamous cell carcinoma and its relationship with microvascular angiogenesis. J Oral Sci Res. (2014) 08:757–9.

[133] Li S Ding Y Zhang X Sun Z. Study on the pro-angiogenic effect of Survivin in the development of oral squamous cell carcinoma. Beijing J Stomatol. (2009) 02:61–4. doi: 10.3969/j.issn.1006-673X.2009.02.001

[134] Wu S Yang X Liu W. Angiogenesis gene chip detection and expression validation in the Malignant transformation of oral leukoplakia. J Pract Stomatol. (2021) 03:337–41. doi: 10.3969/j.issn.1001-3733.2021.03.009

